# APPROACH: Analysis of Proton versus Photon Radiotherapy in Oligodendroglioma and Assessment of Cognitive Health – study protocol paper for a phase III multicentre, open-label randomised controlled trial

**DOI:** 10.1136/bmjopen-2024-097810

**Published:** 2025-02-26

**Authors:** Finbar Slevin, Eleanor Mae Hudson, Florien W Boele, James R Powell, Samantha Noutch, Myfanwy Borland, Sarah Brown, Anna Bruce, Helen Bulbeck, Neil G Burnet, Yen Ching Chang, Rovel Colaco, Stuart Currie, Daniel Egleston, Naomi Fersht, Martin Klein, John Lilley, Matthew Lowe, Elizabeth Miles, Robert D Murray, Daniel J O’Hara, Matthew Norris, Catherine Parbutt, Alexandra Smith, Charlotte Smith, Gillian A Whitfield, Susan Short, Louise Murray

**Affiliations:** 1 Leeds Institute of Medical Research, University of Leeds, Leeds, UK; 2 Leeds Cancer Centre, Leeds Teaching Hospitals NHS Trust, Leeds, UK; 3 Leeds Institute of Clinical Trials Research, University of Leeds, Leeds, UK; 4 Leeds Institute of Health Sciences, University of Leeds, Leeds, UK; 5 Velindre University NHS Trust, Cardiff, UK; 6 The Clatterbridge Cancer Centre NHS Foundation Trust, Liverpool, UK; 7 Brainstrust, Cowes, Isle of Wight, UK; 8 Christie NHS Foundation Trust, Manchester, UK; 9 University College London Hospitals NHS Foundation Trust, London, UK; 10 Department of Radiology, Leeds Teaching Hospitals NHS Trust, Leeds, UK; 11 Medical Psychology, Amsterdam UMC location Vrije Universiteit Amsterdam, Amsterdam, The Netherlands; 12 Mount Vernon Hospital, Northwood, UK; 13 Department of Endocrinology, Leeds Teaching Hospitals NHS Trust, Leeds, UK; 14 St George’s, Lincolnshire Partnership NHS Foundation Trust, Lincoln, Lincolnshire, UK; 15 School of Health and Wellbeing, University of Glasgow, Glasgow, UK; 16 Medicines Management and Pharmacy Services, Leeds Teaching Hospitals NHS Trust, Leeds, UK; 17 Manchester Academic Health Science Centre, Manchester, UK

**Keywords:** Neurological oncology, RADIOTHERAPY, Randomized Controlled Trial

## Abstract

**Introduction:**

Oligodendroglioma (ODG) is a rare type of brain tumour, typically diagnosed in younger adults and associated with prolonged survival following treatment. The current standard of care is maximal safe debulking surgery, radiotherapy (RT) and adjuvant procarbazine, lomustine and vincristine (PCV) chemotherapy. Patients may experience long-term treatment-related toxicities, with RT linked to impairments of neurocognitive function (NCF) and health-related quality of life (HRQoL). With proton beam therapy (PBT), radiation dose falls off sharply beyond the target with reduced normal brain tissue radiation doses compared with photon RT. Therefore, PBT might result in reduced radiation-induced toxicity compared with photon RT.

**Methods and analysis:**

APPROACH is a multicentre open-label phase III randomised controlled trial of PBT versus photon RT in patients with ODG, investigating the impact of PBT on long-term NCF measured using the European Organisation for Research and Treatment of Cancer (EORTC) Core Clinical Trial Battery Composite (CTB COMP). The trial will randomise 246 participants from 18 to 25 UK RT sites, allocated 1:1 to receive PBT or photon RT, with PBT delivered at one of the two UK PBT centres. Participants with grade 2 and grade 3 ODG will receive 54 Gy in 30 fractions and 59.4 Gy in 33 fractions, respectively, followed by 6×6-weekly cycles of PCV chemotherapy. The trial contains staged analyses, with an internal pilot for feasibility of recruitment at 12 months, early assessment of efficacy at 2 years, futility assessment and final primary endpoint comparison of NCF between arms at 5 years. Secondary endpoints include additional NCF, treatment compliance, acute and late toxicities, endocrinopathies, HRQoL, tumour response, progression-free survival and overall survival.

**Ethics and dissemination:**

Ethical approval was obtained from Newcastle North Tyneside REC (reference 22/NE/0232). Final trial results will be published in peer-reviewed journals and adhere to International Committee of Medical Journal Editors (ICMJE) guidelines.

**Trial registration number:**

ISRCTN:13390479.

**STRENGTHS AND LIMITATIONS OF THIS STUDY**

- UK multicentre phase III randomised controlled trial of proton beam therapy versus photon radiotherapy for oligodendroglioma.
- Multistaged analyses will provide continuous assurance of trial processes to ensure successful trial delivery, including preplanned interim analysis to enable early assessment of benefit from proton beam therapy versus photon radiotherapy.
- Trial endpoints and trial design informed by patient and public involvement, with strong emphasis on participant-centred outcome measures.
- Embedded mechanistic component to explore relationships between spatial location of radiation dose to brain substructures and development of neurocognitive toxicities.

## Introduction

 Oligodendroglioma (ODG) is rare, accounting for around 3% of new brain tumour diagnoses with approximately 350 patients diagnosed each year in the UK.^1^ The diagnosis of ODG is determined by the presence of 1p19q chromosomal codeletion and isocitrate dehydrogenase (IDH) mutation,^2^ with histological classification as either WHO grade 2 or 3. ODG typically has a good prognosis, with median survival in excess of 10 years, and median age at diagnosis is approximately 45 years.^3^,^6^ The majority of tumours are located in higher functioning regions of the brain in the frontal or temporal lobes. The current standard of care is maximal safe debulking surgery, radiotherapy (RT) and adjuvant procarbazine, lomustine and vincristine (PCV) chemotherapy.^3 7 8^

Photon RT is typically used to treat ODG, but it may be associated with irreversible long-term toxicities including impairment of neurocognitive function (NCF).^9^,^11^ A recent Cochrane systematic review of long-term neurocognitive changes after photon RT for gliomas concluded that the magnitude of impact on NCF following RT remained uncertain, given the paucity of data and risk of bias inherent in the published literature.^11^ Nevertheless, treatment-related toxicities have the potential to impact on all aspects of life, including daily functioning, work, education, relationships and caring responsibilities, especially given the relatively young age and prolonged survival of many patients with ODG.^12^ In addition, even small deficits in NCF may negatively impact health-related quality of life (HRQoL) and affect activities of daily living.^13^

**Figure 1:**
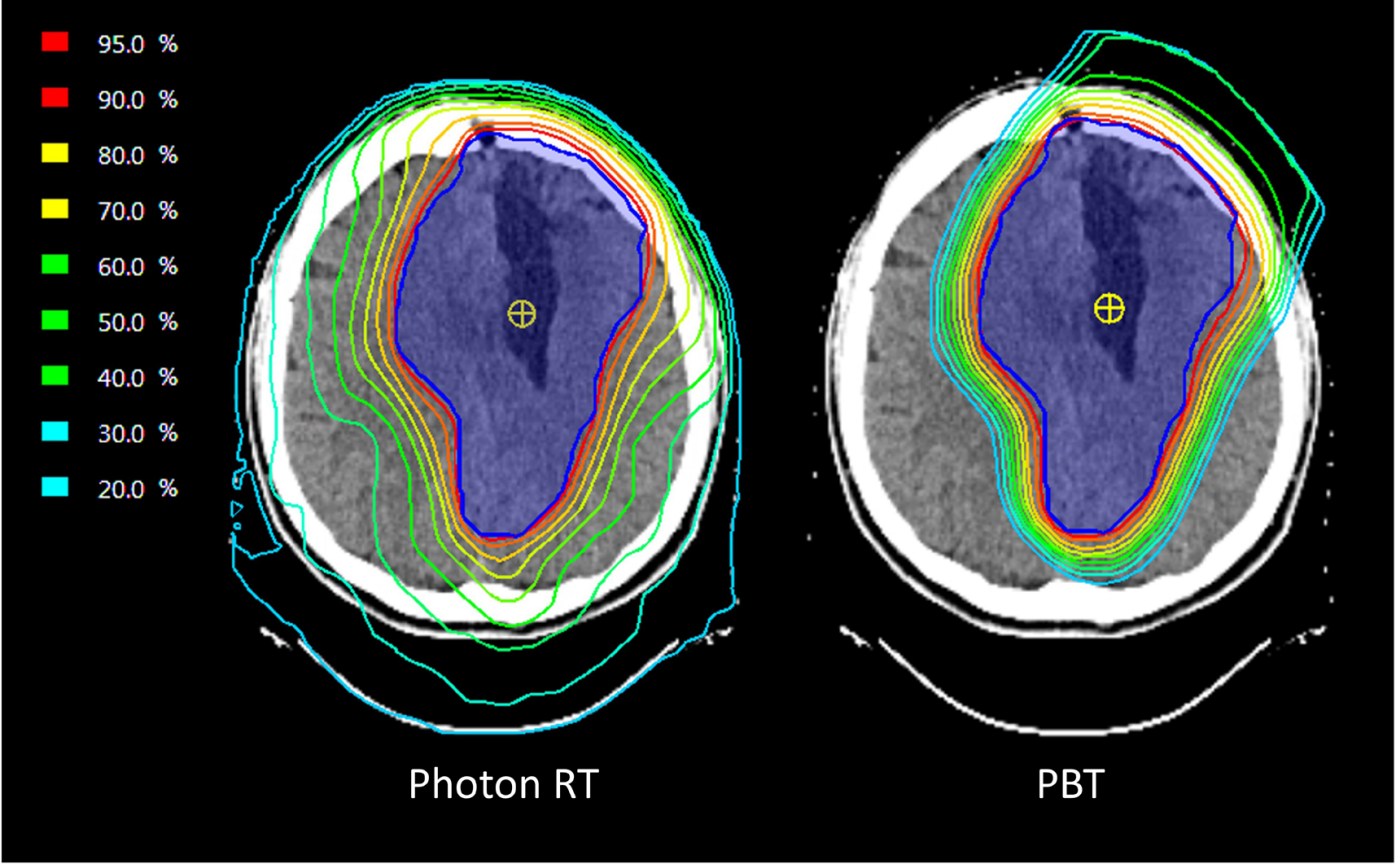
Photon RT and PBT radiotherapy dose distributions in a patient treated for ODG. Planning CT axial images of the brain showing radiation dose distributions for corresponding photon RT and PBT plans. In each plan, the dark blue contour represents the Planning Target Volume (PTV). The isodose lines represent the radiation dose to be delivered as a percentage of the prescription dose, as indicated in the key; warmer colours/higher percentages represent regions of higher dose. Greater dose sparing of normal brain structures outside of the PTV is apparent in the PBT plan. It is currently uncertain if this translates into a clinical improvement in neurocognitive function. ODG, oligodendroglioma; PBT, proton beam therapy; RT, radiotherapy.

It is hypothesised that proton beam therapy (PBT) may result in fewer long-term toxicities compared with photon RT due to relative sparing of surrounding normal brain structures, which could result in less neurocognitive decline. In silico studies in patients with low-grade gliomas have demonstrated dosimetric advantages of PBT (see figure 1), but there remains an absence of high-level evidence that demonstrates clinical improvements in long-term toxicity using this approach.^14^ The outcomes of small, single-arm prospective cohort studies with relatively short follow-up in low-grade glioma suggest that stability in NCF and HRQoL may be achieved after PBT.^15^,^17^ However, there is an urgent need for randomised controlled trials (RCTs) to definitively characterise the clinical benefits of PBT compared with photon RT.

A recent systematic review highlighted the limited current evidence base for PBT, with a need for prospective randomised trials to evaluate its benefits in terms of long-term toxicities and patient-reported outcomes.^18^ PBT is now available in two UK National Health Service (NHS) centres (The Christie NHS Foundation Trust and University College London NHS Foundation Trust), which provides an opportunity to develop a practice-changing evidence base for PBT.

## Methods and analysis

### Study design

APPROACH: Analysis of Proton versus Photon Radiotherapy in Oligodendroglioma and Assessment of Cognitive Health is a multicentre open-label parallel group phase III RCT of PBT versus photon RT in patients with ODG. The aim of the study is to determine whether participants treated with PBT demonstrate better NCF compared with those treated with photon RT. A mechanistic component will investigate the relationship between RT dosimetry in specific brain regions and impairments in NCF.

**Figure 2:**
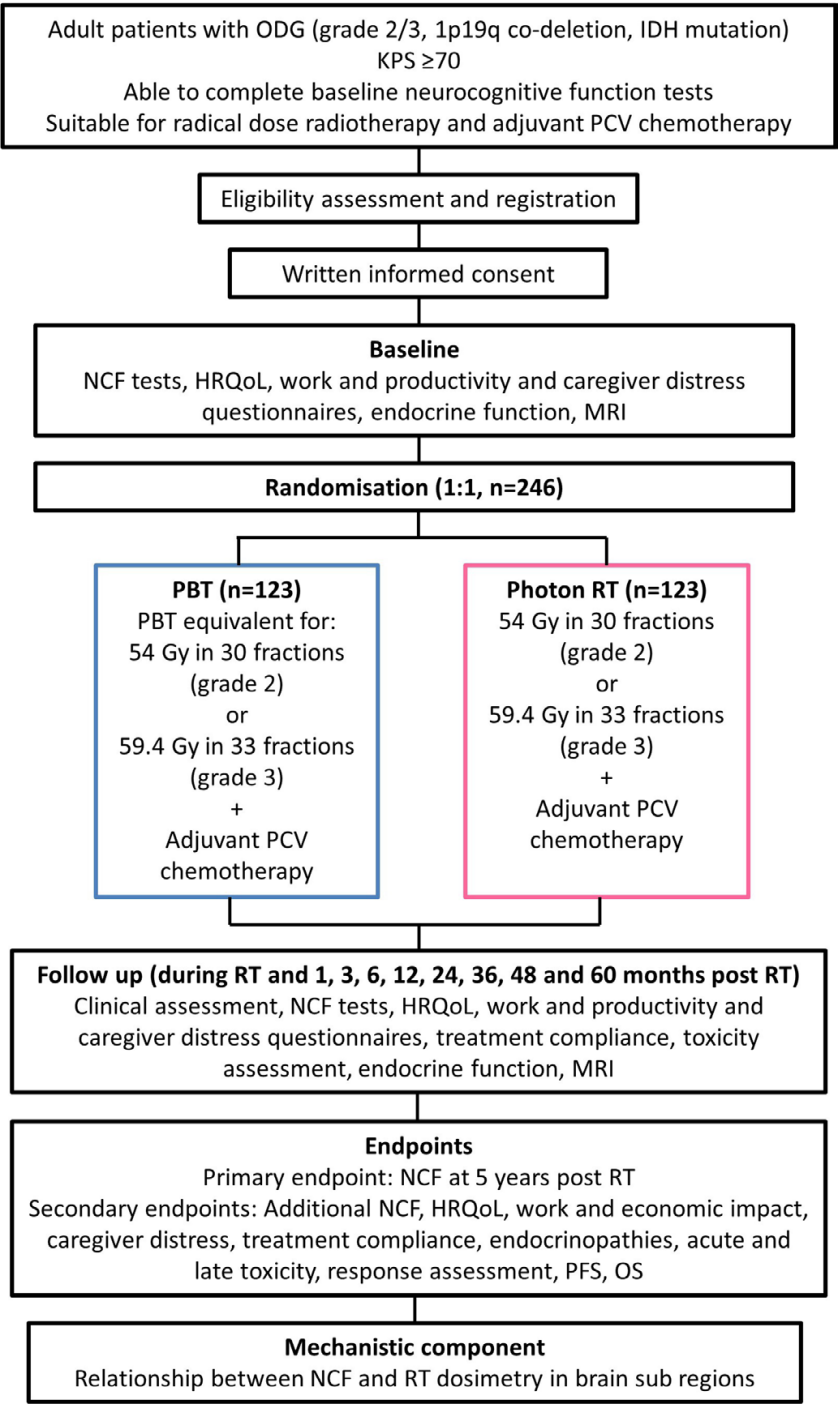
Trial schema. HRQoL, health-related quality of life; IDH, isocitrate dehydrogenase; KPS, Karnofsky Performance Status; NCF, neurocognitive function; ODG, oligodendroglioma; OS, overall survival; PBT, proton beam therapy; PCV, procarbazine, lomustine and vincristine; PFS, progression-free survival; RT, radiotherapy.

Participants are patients with ODG who require RT, followed by adjuvant PCV chemotherapy, and who meet all eligibility criteria for the study (inclusion and exclusion criteria are listed in table 1), and their caregivers. Potential participants will be identified at local neuro-oncology multidisciplinary team meetings, approached at outpatient clinics and provided with the study Participant Information Sheet. Patients will be given time to consider participation. Written informed consent will be obtained. Participants will be randomised 1:1 to PBT or photon RT from 18 to 25 UK RT centres, with PBT delivered in one of the two NHS proton centres, depending on geographical location with consideration for availability and photon RT delivered in the local RT centre. For participants allocated to PBT, the NHS will provide accommodation for preassessment visits and for the duration of PBT for the participant and one partner/caregiver. Following RT, all participants will receive adjuvant PCV chemotherapy at their local site. Follow-up will also be at local sites as per standard of care. Trial-specific participant assessments are summarised in table 2. The trial schema is shown in figure 2.

**Table 1:** Inclusion and exclusion criteria

Inclusion criteria	Exclusion criteria
Histologically confirmed grade 2/3 ODG, 1p19q codeletion and IDH mutation	Leptomeningeal, spinal or infratentorial disease
Randomisation within 28 days of MRI which supports decision that RT is required at that point in time—outside of 28 days, an updated MRI is required	Prior cranial or head and neck RT
Aged 25 or over at start of RT	Previous chemotherapy for ODG. The use of vorasidenib or similar IDH inhibitors prior to radiotherapy, should these become available in the UK, will not render the patient ineligible
KPS≥70	Comorbid neurological condition(s) influencing NCF
Adequate wound healing/recovery following surgery (if applicable)	Contraindication to MRI or gadolinium
Able to provide study-specific written informed consent	Severe active comorbidity making patient unsuitable for radical RT and/or adjuvant chemotherapy
Able to complete baseline NCF testing in English	Any recognised genetic syndrome causing sensitivity to RT
Adequate haematological, renal and hepatic function for PCV chemotherapy	Contraindication to procarbazine, lomustine or vincristine, including coeliac disease and rare hereditary conditions of galactose intolerance, total lactase deficiency or glucosegalactose malabsorption
Able to swallow oral medication	Prior invasive malignancy, unless disease-free interval ≥3 years
Participants born female of childbearing potential must agree to be pregnancy screened prior to entering the trial, provide a negative pregnancy result within 7 days prior to randomisation and agree to use medically acceptable methods of contraception during RT, between the end of RT and start of adjuvant chemotherapy, during chemotherapy and for 6 months following the end of chemotherapy	Pregnancy or breastfeeding
Fertile participants born male must agree to use medically acceptable methods of contraception during RT, between the end of RT and start of adjuvant chemotherapy, during chemotherapy and for 6 months following the end of chemotherapy	Participant unable or unwilling to attend for follow-up
	Recognised genetic syndrome causing sensitivity to radiotherapy

IDHisocitrate dehydrogenaseKPSKarnofsky Performance StatusNCFneurocognitive functionODGoligodendrogliomaPCVprocarbazine, lomustine and vincristine chemotherapyRTradiotherapy

**Table 2:** Schedule of assessments

	Baseline/pretreatment			Treatment	Post-RT follow-up							
	Eligibility/preregistration	Prerandomisation	Pre-RT	Weekly RT	1 month	3 months	6 months	1 year	2 years	3 years	4 years	5 years
Medical history	X											
Clinical assessment	X			X	X	X	X	X	X	X	X	X
Pregnancy test	X	X	X		X	X	X					
Informed consent	X											
MRI scan	X		X			X	X	X	X	X	X	X
Radiotherapy planning CT scan			X									
PBT or photon RT				X								
PCV chemotherapy						6×6-weekly cycles of adjuvant PCV chemotherapy						
Neurocognitive function tests*		X			X			X	X	X	X	X
CTCAE toxicity assessment		X		X	X	X	X	X	X	X	X	X
Endocrine assessments			X				X	X	X	X	X	X
HRQoL†		X		X‡	X	X	X	X	X	X	X	X
Work and economic impact§		X		X‡	X	X	X	X	X	X	X	X
Caregiver distress¶		X		X*‡	X	X	X	X	X	X	X	X

* EORTC core CTB COMP and CNS Vital Signs test battery.

† EORTC QLQ-C30 and BN20, EQ-5D-5L, MFI, HADS.

‡ Once in the final week of RT.

§ WPAI: General Health Questionnaire and health resource use questionnaire.

¶ Caregiver needs screen.

CNScentral nervous systemCTCAECommon Terminology Criteria for Adverse EventsEORTC CTB COMPEuropean Organisation for Research and Treatment of Cancer Core Clinical Trial Battery CompositeEORTC QLQ-C30 and BN20European Organisation for Research and Treatment of Cancer Quality of Life Questionnaire and Brain Neoplasm QuestionnaireEQ-5D-5LEuroQol-5 Dimension-5 Response-Level QuestionnaireHADSHospital and Anxiety Depression ScaleHRQoLhealth-related quality of lifeMFIMultidimensional Fatigue AssessmentPBTproton beam therapyPCVprocarbazine, lomustine and vincristineRTradiotherapyWPAIWork Productivity and Activity Impairment

The main trial analyses will be staged:

- Stage 1: assessment of feasibility of recruitment (first 12 months)
- Stage 2: efficacy interim analysis of NCF (all participants at 2 years)
- Stage 3: futility interim analysis of NCF (50% of participants at 5 years)
- Stage 4: final analysis of NCF and secondary endpoints (all participants at 5 years)

The original planned start and end dates for the trial are 1 February 2021 to 17 July 2031. Recruitment commenced on 17 January 2024.

See ‘Statistical considerations’ section for further detail.

### Radiotherapy

The *APPROACH RT guidelines* provide full details on immobilisation, planning image acquisition, target volume and organ at risk contouring, treatment planning, treatment delivery and quality assurance.

RT will ideally start within 6 weeks of randomisation (and must start within 10 weeks). A dedicated MRI scan to assist in RT planning is mandated. The immediate postoperative MRI must not be used for this purpose, as post-operative changes may not have had sufficient time to resolve.

Total RT dose will be 54 Gy (RBE, relative biological effectiveness) in 30 daily 1.8 Gy fractions over approximately 6 weeks for grade 2 ODG and 59.4 Gy (RBE) in 33 daily 1.8 Gy fractions over approximately 6.5 weeks for grade 3 ODG. RBE-weighted dose in units of Gray (Gy (RBE)) will be used to describe the product of the absorbed dose and the RBE. The RBE will be interpreted as 1.1 for PBT and 1.0 for photon RT.

RT will be delivered as an outpatient on weekdays. Photon RT will be delivered using intensity-modulated RT or volumetric-modulated arc therapy. PBT will be delivered using pencil beam scanning, typically performed with single-field optimisation. Each participant treated using PBT will have an equivalent photon RT plan produced for use in the event of PBT machine problems.

For photon RT, local practice for treatment verification will be followed. For PBT, daily online image-guided verification with positional correction using 2D kV imaging or cone beam CT will be performed.

### Chemotherapy

Adjuvant PCV chemotherapy is per standard of care, which in the UK is usually according to the BR12 trial schedule.^19^ Chemotherapy should start within 4–8 weeks of completion of RT. Initial doses (in the absence of renal or hepatic impairment) will be as follows:

- Procarbazine 100 mg/m^2^, with dose banding/capping as per the institution’s usual practice one time per day on days 1–10 or days 2–11, orally, on a 42 day cycle, for up to six cycles.
- Lomustine 100 mg/m^2^, with dose banding/capping as per the institution’s usual practice day 1, orally, on a 42 day cycle, for up to six cycles.
- Vincristine 1.4–1.5 mg/m^2^ (or flat dose of 2 mg if this is usual institutional practice), with dose banding/capping as per the institution’s usual practice, intravenously, day 1, on a 42 day cycle for up to six cycles.

Suggested dose modifications and reductions are provided in the APPROACH trial protocol regarding PCV in the presence of haematological, pulmonary or neurological toxicity or renal or hepatic dysfunction.

### Endpoints

#### Primary endpoint

The primary outcome is NCF assessed at baseline and at 1, 12, 24, 36, 48 and 60 months post-RT. NCF will be measured by the paper-based European Organisation for Research and Treatment of Cancer (EORTC) Core Clinical Trial Battery Composite (CTB COMP).^20 21^ CTB COMP evaluates processing speed, verbal memory and executive functioning.

The CTB COMP consists of the following tests, yielding six measures:

- Hopkins Verbal Learning Test-Revised (HVLT-R) consists of three parts: free recall, delayed recall and delayed recognition. It measures various aspects of verbal learning and memory, namely storage of verbal information as well as active and passive retrieval of this information.
- Trail Making Test (TMT part A and TMT part B). Part A indexes visual-motor scanning speed, while part B assesses executive functioning.
- Controlled Oral Word Association test (COWA) measures expressive language.

The primary endpoint will be the difference in NCF scores, measured by CTB COMP, between the PBT and photon RT arms at 5 years post-RT.

#### Secondary endpoints

Secondary endpoints are shown in table 3.

**Table 3:** Secondary endpoints

Endpoint	Definition/measurement
Additional NCF tests	Computer-based CNS Vital Signs platform, which evaluates attention, psychomotor speed, verbal/visual memory and aspects of executive functioning^44^
Caregiver distress	Caregiver needs screen
Work and economic impact (participants and carers)	WPAI: General Health Questionnaire, and Health Resource Use Questionnaire
Treatment compliance	Total dose of RT delivered; overall treatment time; details of any interruptions to RT and reasons; delivery of any fractions using photon RT instead of PBT; number of chemotherapy cycles; chemotherapy doses delivered; details of any modifications and reasons
Endocrinopathies	Static/dynamic testing of the following on blood samples: GH/IGF-1 FSH/LH Testosterone and SHBG (male participants) Oestradiol (female participants) Cortisol Free T3/T4, TSH Prolactin
Safety and toxicity	Acute (≤3 months post-RT) and late (>3 months post-RT) toxicities measured using CTCAE V.5.0.
HRQoL	EORTC QLQ-C30 and BN20, EQ-5D-5L, MFI and HADS.
Tumour response assessment	MRI brain performed with the following sequences: • T2 • FLAIR • DWI • T1 pregadolinium T1 postgadolinium Evaluation based on RANO criteria.
PFS	Time from randomisation to the date of the first-documented evidence of progression or death from any cause.
OS	Time from randomisation to the date of death from any cause.

CNScentral nervous systemCTCAECommon Terminology Criteria for Adverse EventsDWIdiffusion-weighted imagingEORTC QLQ-C30 and BN20European Organisation for Research and Treatment of Cancer Quality of Life Questionnaire and Brain Neoplasm QuestionnaireEQ-5D-5LEuroQol-5 Dimension-5 Response-Level QuestionnaireFLAIRfluid-attenuated inversion recoveryFSHfollicle stimulating hormoneGHgrowth hormoneHADSHospital Anxiety and Depression ScaleHRQoLhealth-related quality of lifeIGF-1insulin like growth factor 1LHleutinising hormoneMFIMultidimensional Fatigue AssessmentNCFneurocognitive functionOSoverall survivalPBTproton beam therapyPFSprogression-free survivalRANOresponse assessment in neuro-oncologyRTradiotherapySHBGsex hormone binding globulinTSHthyroid stimulating hormoneWPAIWork Productivity and Activity Impairment

### Statistical considerations

#### Sample size

The primary endpoint of NCF at 5 years will be measured by calculating the mean of standardised z-scores from the HVLT-R, TMT-A/B and COWA components of CTB COMP. A Cohen’s d of 0.5 is regarded as a moderate effect size, which is considered to be clinically relevant given that even small deteriorations in NCF are likely to be impactful in this patient population.^22^ This CTB COMP score is also being used as the primary endpoint measure in the ongoing US NRG-BN005 trial of PBT versus photon RT for cognitive preservation in IDH mutant grade 2/3 glioma (Clinicaltrials.gov NCT03180502). Based on a two-sample t-test with 5% two-sided significance and 90% power, 172 participants (86 per arm) are required to detect an effect size of 0.5. Assuming 30% loss to follow-up at 5 years, 123 participants will be required per arm. The required sample size is therefore 246 participants, to be recruited over 3.5 years. The study is designed with an intermediate and final primary endpoint, whereby a significant positive treatment effect on either endpoint would warrant change in practice. Following the Food and Drug Administration (FDA) guidance on multiple endpoints in clinical trials, the family-wise error rate of 0.05 will be preserved using the fall-back method,^23^ with alpha of 0.01 allocated to the intermediate and 0.04 allocated to the final primary endpoint. Should a significant effect be observed on the intermediate endpoint, the full alpha of 0.05 will be used for the final primary endpoint analysis. Irrespective of the early interim assessment outcome, the trial will continue. Assuming the same sample size calculation, using a type I error of 0.04 (5 years) would provide ~89% power. At the 2 year time point, assuming a smaller dropout rate of 10%, a type I error of 0.01 would provide ~87% power.

#### Randomisation

A computer-generated minimisation programme that incorporates a random element will be used to ensure the treatment groups are well balanced for the following factors:

- Histological tumour grade (2 or 3)
- Tumour size (<5 cm or ≥5 cm)
- Extent of most recent surgery (biopsy only, subtotal resection or gross total resection)
- Randomising site
- Sex

Randomisation will be performed centrally via University of Leeds Clinical Trials Research Unit (CTRU) automated 24-hour randomisation system. The registration and randomisation process will be instigated by onsite research staff; participant consent form must be attained prior to registration (see online supplemental material).

#### Statistical analyses

The trial will be analysed in stages. A detailed statistical analysis will be written before any analysis is undertaken, including interim and final. Statistical analysis will be conducted by the CTRU.

Stage 1: the feasibility of recruitment (numbers of sites opened and participants recruited) will be assessed after the first 12 months. Prespecified traffic light targets will guide assessment of stage 1.^24^ If ≥57 participants are recruited (green), the trial will continue immediately to stage 2. If 37–56 participants (amber) are recruited, barriers to recruitment and remedial action will be explored to inform future trial feasibility. If fewer than 37 participants are recruited (red), a full review of the feasibility of the trial will be undertaken with potential trial closure.

Stage 2: interim analysis of 2 year NCF once all participants have reached ≥2 years follow-up. Stage 2 will analyse early evidence of efficacy, defined by a clinically relevant difference in NCF (Cohen’s d effect size of ≥0.5) between participants treated with PBT and photon RT, using a 1% significance level to account for multiple testing.^25^ The trial will continue to stage 3, regardless of stage 2 results.

Stage 3: further interim analysis of NCF once 50% of participants have reached 5 years follow-up to assess futility. Conditional power will be used to evaluate the probability of achieving a statistically significant result for the final analysis of the primary endpoint, based on accumulated data. A predefined non-binding stopping boundary will be determined in collaboration with the Data Monitoring and Ethics Committee (DMEC) prior to the analysis.^26^

Stage 4: final analysis of primary endpoint of NCF at 5 years and all secondary endpoints.

##### Primary endpoint

The primary endpoint will be assessed on an intention to treat population, including all randomised participants, in the treatment arm they were randomised to. Summary statistics will be presented for NCF scores at each timepoint (baseline and at 1, 12, 24, 36, 48 and 60 months post-RT). A mixed-effects repeated measures model will be used to evaluate differences in mean NCF scores between the two treatment arms. The model will adjust for minimisation factors, baseline NCF score, timepoint, treatment group and treatment group by timepoint interaction as fixed effects. Participant and participant time interaction will be fitted as random effects, as appropriate. The final estimated treatment effect will be reported with 95% CIs and associated significance level. Additional 96% (4% level) CIs will be reported if there is no significant effect at the interim analysis (stage 2). The effect size, accounting for SD, will also be estimated in line with the sample size assumptions.

##### Secondary endpoints

Summary statistics by treatment arm at each timepoint will be presented for additional tests of NCF, HRQoL, caregiver distress, work and economic impact, treatment compliance, endocrinopathy and response assessment. Similar mixed-effects repeated measures models to those used for the primary endpoint will be used to compare HRQoL and additional NCF tests between treatment arms, adjusted for minimisation factors, relevant clinical characteristics and baseline HRQoL.

The numbers and proportions of participants experiencing each Common Terminology Criteria for Adverse Events (CTCAE) V.5.0 acute and late toxicity grade will be summarised.^27^ Safety data will be summarised using the safety population, with participants summarised according to the treatment they received. The proportion of participants experiencing serious adverse reactions and related unexpected serious adverse events will also be summarised.

Overall survival and progression-free survival will be analysed using Cox proportional hazards models. Kaplan-Meier and time to event estimates with 95% CIs will be presented.

## Mechanistic component

It is hypothesised that there are definable RT dose–response relationships for specific structures/regions within the brain that are associated with impairments in NCF.^28^ Neurocognition is complex, and it relies on extensive, interconnecting networks of structures. The dose–response relationships for many of these regions and networks remain undefined.^28 29^ The APPROACH trial is an important opportunity to compare RT dosimetry with prospectively collected NCF data. Two approaches will be used as part of this work.

In the first approach, segmentation of the following structures will be performed on coregistered planning CT and MRI scans, based on existing atlases:^2330^,^33^ hippocampi (regions of neurogenesis and role in memory), temporal lobes (language, hearing and memory), cerebellum (attention, motor functioning), corpus callosum (processing speed), subventricular zones (neurogenesis), frontal white matter, anterior cingulate gyrus and frontal pole (executive functioning, memory, personality/behaviour and attention), precentral gyrus (primary motor cortex), occipital lobes (vision) and parietal lobes (sensation, spatial awareness). Dosimetric data will be exported from RT plans and corrected for fractionation to the equivalent dose in 2 Gy fractions (EQD2). These data will be compared with NCF data to identify relevant dose impairment thresholds for brain structures using methods similar to those used by Gondi *et al*, with consideration given to multiple hypothesis testing.^34^

In the second approach, lesion symptom mapping will be performed. This is a method for relating a specific region within the brain to a particular function and has been used in the setting of stroke and trauma.^35^ Similar to the method used by Habets *et al*, RT doses will be segmented on planning CT/MRI scans in dose bands in EQD2 and resection cavities/tumour will be segmented and subtracted to produce a map of normal tissue doses.^36^ Individual normal tissue dose location maps (DLM) will be registered to a standard 3D brain template and grouped together to create probability maps for the presence of dose in each voxel. For each neurocognitive domain and each dose band, we will compare DLMs at voxel level between participants with and without neurocognitive impairment at 5 years, with consideration of multiple hypothesis testing.

The mechanistic component may identify particularly sensitive brain subregions and/or interconnecting neural networks, the dose to which could be constrained during RT planning. It could also highlight scenarios where the normal tissue sparing properties of PBT would be particularly helpful. Where normal tissue doses to particular brain regions cannot be minimised, for example due to tumour location, improved understanding of relationships between RT dosimetry and impairment of NCF may permit more informed discussions regarding potential benefits and risks of treatment. It could also inform strategies to provide targeted neurorehabilitation of specific RT-related neurocognitive toxicities.

## Trial organisation

Trial coordination, data management and statistical aspects of APPROACH will be managed and conducted by the CTRU. Trial organisation will be in line with CTRU standard operating procedures and aligned with principles of Good Clinical Practice.

### Data collection and management

Data collection for APPROACH will be largely remote data entry, with some elements recorded on paper including serious safety events, NCF, and, optionally, participant-reported outcome measures (PROMs) questionnaires. These will be sent to and entered by the CTRU. PROMs can also be completed online using REDCap. Participant data will be recorded on trial-specific databases. Each database includes automatic validations and checking procedures. Where possible, data will be collected from withdrawn participants who do not withdraw consent from further data collection.

Data collected during the trial will be kept confidential during and after the trial and stored securely at the CTRU. Only the trial team and key members of CTRU staff will have access to the full trial data. At the end of the trial, data will be archived in line with the sponsor’s procedures for a minimum of 15 years. After the final trial results publication, researchers may request access to data from the Trial Management Group (TMG) and CTRU.

### Quality assurance

#### Radiotherapy quality assurance

RT quality assurance will be conducted by the UK National RT Trials Quality Assurance (RTTQA) Group, to include pretrial and on-trial case review. Benchmarking cases will be completed by all participating sites, with prospective review of the first case and retrospective review of subsequent cases. RT dosimetry and treatment data will be collected for all participants.

#### Site initiation and training

All essential documentation and electronic site initiation processes must be complete before a site can open to recruitment. Key members of staff at local sites will be responsible for performing NCF tests on participants and therefore must also complete NCF training. Completion of NCF test booklets will also be reviewed at intervals by the trial team, and feedback provided. Refresher training is also required at specific intervals.

#### Trial monitoring

APPROACH will be monitored by a multidisciplinary TMG including clinical trial, neuro-oncology and PBT expertise and patient and public involvement (PPI) representation. The independent DMEC will meet at least annually. This committee will review unblinded data and oversee the safety and integrity of the trial. The Trial Steering Committee (TSC) will receive advice from the DMEC on trial safety and continuation recommendations, and also includes PPI representation. DMEC and TSC charters define roles and responsibilities for each committee member.

## Patient and public involvement

PPI has been central to the development of APPROACH. A focus group with 15 patients previously treated with RT for ODG and their caregivers was held to inform the design of APPROACH.^37^ Discussions focused on aspects of study design and potential impact of travel and temporary relocation for participants randomised to PBT. Importantly, feedback from focus group attendees resulted in modification of HRQoL assessments in APPROACH, to ensure that greater information would be obtained regarding fatigue and daily well-being, and inclusion of caregivers’ perspectives.

## Ethics and dissemination

Ethical approval was obtained from Newcastle North Tyneside REC (reference 22/NE/0232). The trial was registered with ISRCTN (13390479) on 10 March 2023. APPROACH is not a trial of an investigational medicinal product (non-CTIMP). The APPROACH trial currently adheres to protocol V.3 (17 April 2024). All protocol amendments will be submitted to the REC and communicated with local sites and the PBT centres.

Data from staged analyses may be reported with approval of the trial monitoring committees. All trial manuscripts, including final trial results, will be published open access in peer-reviewed journals and adhere to International Committee of Medical Journal Editors (ICMJE) guidelines.

## Discussion

APPROACH will provide prospective, randomised evidence of whether PBT provides benefit compared with photon RT for patients with ODG with regard to RT-related impairments in NCF. It will provide important insights into HRQoL, caregiver distress, effects on work and other activities of daily living and long-term toxicities associated with PBT compared with photon RT for a population of patients who tend to be of younger age, with work and caring responsibilities and typically experience long-term survival. The mechanistic component of the trial will explore relationships between RT dosimetry in specific brain subregions and development of impairments in NCF. This work has potential to identify sensitive brain structures and interconnecting neural networks where dose could be constrained during RT planning with the goal of reducing impairments in NCF.

APPROACH is the result of close collaboration between multidisciplinary teams of clinicians, scientists, methodologists, clinical trialists, statisticians and PPI representatives, who have worked with national research advisory groups including the previously funded National Cancer Research Institute (NCRI) Clinical and Translational Radiotherapy Working Group (CTRad) PBT Clinical Trial Strategy Group to design, fund and deliver the trial. NCRI CTRad organised national meetings to develop PBT clinical trials, including APPROACH, as well as the TORPEdO and PARABLE trials in oropharynx and breast cancers, respectively.^38 39^ APPROACH is aligned with the eight-point framework developed by CTRad’s PBT Clinical Trial Strategy Group, and it demonstrates the strength of this co-ordinated approach to the development of RT clinical trials in the UK.^40^ Alongside trials of PBT for other disease indications currently in progress, APPROACH will provide high-level evidence of the impact of PBT on long-term RT toxicities in ODG and identify which patients are most likely to benefit compared with photon RT.^38 39 41^ In addition, these data may be relevant for other patient cohorts, including meningioma and other good-prognosis non-OGD glioma subtypes. Building the evidence base for PBT is essential, given the significant capital investment funding required in the set-up of PBT centres and increased costs associated with this treatment compared with photon RT.^40^

Two other RCTs comparing PBT with photon RT for glioma with primary endpoints of NCF are currently in progress. NRG-BN005 is a US phase II trial of 120 participants treated using PBT or photon RT followed by adjuvant temozolomide chemotherapy (NCT03180502). The primary endpoint is NCF at 10 years, also measured using CTB COMP, as in APPROACH. GliProPh is a German trial of 80 participants with a primary endpoint of NCF at 3 years (German Clinical Trials Register DRKS00015160). These trials are investigating PBT in patients with IDH mutant grade 2/3 gliomas, an overlapping, but not identical population to that included in APPROACH (including patients with tumours without 1p19q codeletion, who have a poorer prognosis). The findings of these trials will therefore not be directly applicable to patients with ODG, underlying the importance of APPROACH in delivering the evidence for PBT in this specific subgroup of good prognosis glioma. Given the alignment in terms of neurocognitive tests and effect size used for the primary endpoint in each trial, this may present the opportunity to work collaboratively with the US team leading NRG-BN005, in order to develop the evidence base for PBT across molecular pathologies and treatment regimens. Similarities between APPROACH and NRG-BN005 present a potential opportunity to combine data in a future meta-analysis.

While APPROACH will provide NCF data up to 5 years post-RT, previous data suggest prevalence and severity of impairments in NCF may continue to increase beyond 5 years.^42^ This highlights the importance of determining whether NCF is better for participants treated with PBT compared with photon RT. Additionally, the PBT centres’ Proton Clinical Outcome Units will continue to collect long-term clinical and patient-reported outcome data for patients treated with PBT, which presents an opportunity to gain valuable insights into the impact of PBT for participants in APPROACH beyond the 5 year timepoint.^43^

APPROACH will deliver high-quality evidence of the impact of PBT on NCF and other long-term toxicities in patients with ODG, provide insight into HRQoL through participant-reported and caregiver-reported outcome measures and improve understanding of the relationships between RT dosimetry in specific brain regions and impairments of NCF.

## supplementary material

10.1136/bmjopen-2024-097810 online supplemental file 1
