# Supplementary material for: APPROACH: Analysis of Proton versus Photon Radiotherapy in Oligodendroglioma and Assessment of Cognitive Health – study protocol paper for a phase III multicentre, open-label randomised controlled trial
Source: BMJ Open. 2025 Feb 26;15(2):e097810. doi: 10.1136/bmjopen-2024-097810 (PMC11865786; doi:10.1136/bmjopen-2024-097810)
Supplement: online supplemental file 1 [file bmjopen-15-2-s001.docx]

## Table S1: Survey questions

| **Question relating to harm**   - Q1. In the last three years, have you been physically or emotionally harmed by your NHS treatment or care, or the lack of it? ^1^ - Q2. Where did the harm first happen? - Q3. … what impact, if any, did the harm have on your physical health? - Q4. … what impact, if any, did the harm have on your emotional well-being or mental health? - Q5. … what impact, if any, did the harm have on your usual activities (for example, work, education, social activities, or caring responsibilities)? - Q6. Which of the following actions, if any, did you take as a result of being harmed? - Q7. How well, or not, did you think your formal complaint was handled? - Q8. How helpful, or not, did you find PALS, the Patient Advice and Liaison Service? - Q9. … what response did you want from the healthcare provider where the harm happened? - Q10. What is the main reason, if any at all, you didn’t pursue a legal claim for financial compensation?   **Demographics**   - Age (18-24; 25-34; 35-44; 45-54; 55-64; 7: 65+ - Sex (male and female) - Geographical region (East Midlands; Eastern; London; North East; North West; Scotland; South East; South West; Wales; West Midlands; and Yorkshire and Humber) - Employment (Full-time employed; Part-time employed; Self-employed; Economically inactive (students, retired people, home keeper); Not working (unemployed and seeking work, not in paid work for other reasons); Disabled/ long-term illness (not working). - Self-reported ethnicity (White British; White Other; Mixed; Asian/ Asian British, Black/ Black British; and Other) ^2^ - Social grade - National Readership Survey Classification A (highest grade) to E (lowest grade) <https://nrs.co.uk/nrs-print/lifestyle-and-classification-data/social-grade/> |
| --- |

^1^ The previous question on the rate of harm (Gray et al (8) and Fenn et al (19)) was “Over the last three years, have you suffered any illness, injury or impairment that in your opinion was caused by your medical treatment or care?”

^2^ Categories computed from 16 response options used by IPSOS and by the Office of National Statistics. The 16 response options are: White British (‘White British); White Irish, White Gypsy /Traveller and White Other (‘White Other’); Mixed White/Black Caribbean, Mixed White/Black African, Mixed White And Asian and Mixed Other (‘Mixed’); Asian Indian, Asian Pakistani, Asian Bangladeshi, Asian Chinese, and Asian Other (Asian/ Asian British); Black African, Black Caribbean, and Black Other (Black/ Black British); Arab and Other (Other)

## Table S2: Cognitive Interviews

| **Recruitment**  Cognitive interviews, which were conducted over the telephone with eight women and four men (age range 47-73 years; 10 White British; 2 working, 3 retired, 3 permanently sick or disabled, 1 doing something else and 3 unknown); and 11 of whom had experience of harm and 1 was the spouse of someone with experience of harm. The 12 participants were recruited via two patient and public involvement (PPI) groups and snowballing. People were eligible to participate if they were aged ≥18 years and reported physical or emotional harm through the NHS.  **Process**  A concurrent probing method,^1^ was used to evaluate the questionnaire, with a researcher reading each question to the participant and the participant providing their response. This was followed by a series of cognitive probes to assess comprehension, ability to retrieve the requested information, certainty of response, and the suitability and breadth of response options. The interviews were audio-recorded. Findings for each round were summarised in a matrix, discussed by the research team. Where necessary, revisions to a question were made prior to the next round. After three rounds, key issues had been identified and rectified. |
| --- |

^1^ Willis, G.B. Analysis of the cognitive interview in questionnaire design. New York: Oxford University Press

## Table S3: Sociodemographic factors for the total sample (n=10,064) (weighted analysis)

|  |  | Total n | % |
| --- | --- | --- | --- |
| **Demographics** | Groups |  |  |
| **Age (years)** | 18-24 | 1126 | 11.2 |
|  | 25-34 | 1725 | 17.1 |
|  | 35-44 | 1604 | 15.9 |
|  | 45-54 | 1705 | 16.9 |
|  | 55-64 | 1537 | 15.3 |
|  | 65+ | 2367 | 23.5 |
| **Sex** | Male | 4906 | 48.8 |
|  | Female | 5079 | 50.5 |
| **Ethnicity** | White British | 8074 | 80.2 |
|  | White Other | 591 | 5.9 |
|  | Mixed | 251 | 2.5 |
|  | Asian | 520 | 5.2 |
|  | Black | 421 | 4.2 |
|  | Other | 128 | 1.3 |
| **Employment** ^1^ | Full-time | 3937 | 39.3 |
|  | Part-time employed | 1062 | 10.6 |
|  | Self-employed | 945 | 9.4 |
|  | Economically inactive | 3251 | 32.4 |
|  | Not working | 463 | 4.6 |
|  | Disability or long-term illness | 369 | 3.7 |
| **Region** | East Midlands | 744 | 7.4 |
|  | Eastern | 970 | 9.6 |
|  | London | 1376 | 13.7 |
|  | North East | 421 | 4.2 |
|  | North West | 1148 | 11.4 |
|  | Scotland | 876 | 8.7 |
|  | South East | 1410 | 14.0 |
|  | South West | 891 | 8.9 |
|  | Wales | 480 | 4.8 |
|  | West Midlands | 892 | 8.9 |
|  | Yorkshire and Humberside | 853 | 8.5 |
| **Education** | GCSE/O-level | 1402 | 13.9 |
|  | Vocational | 629 | 6.3 |
|  | A-level or equivalent | 1979 | 19.8 |
|  | University degree or equivalent | 2595 | 25.9 |
|  | Postgraduate degree or equivalent | 1280 | 12.8 |
|  | Other | 683 | 6.8 |
|  | No formal qualifications | 1454 | 14.5 |

## Table S3 (continued): Sociodemographic factors for the total sample (n=10,064) (weighted analysis)

|  |  | Total n | % |
| --- | --- | --- | --- |
| **Social grade ^2^** | A | 492 | 4.9 |
|  | B | 2178 | 21.6 |
|  | C1 | 2477 | 24.6 |
|  | C2 | 2052 | 20.4 |
|  | D | 1436 | 14.3 |
|  | E | 925 | 9.2 |
|  | Unknown | 506 | 5.0 |

^1^ Full-time – employed 30+ hours; Part-time – employed 29 hours or less; Economically inactive – full time students, still at school, retired or not in paid work due to long-term illness or disability; Not working – Unemployed and seeking work, Not in paid work for other reasons, or not working – housewife

^2^ Social grade: National Readership Survey Classification A (highest grade) to E (lowest grade) <https://nrs.co.uk/nrs-print/lifestyle-and-classification-data/social-grade/>

## Graph S1: Distribution of predicted risk of harm from the full model, stratified by employment categories.

Key: Disabl/ill = Not working due to disability or long-term illness

## Table S4: Location where the harm first occurred

|  | **n** | **%** |
| --- | --- | --- |
| **Lack of access to care** | 362 | 36.7 |
| **Hospital – all** | 274 | 27.7 |
| Hospital – inpatient | 120 | 12.2 |
| Hospital - outpatient | 92 | 9.3 |
| Hospital - day surgery | 37 | 3.7 |
| Hospital - Other | 25 | 2.5 |
| **GP surgery** | 205 | 20.7 |
| **Home** | 61 | 6.2 |
| **Other** | 52 | 5.3 |
| **Dental surgery** | 22 | 2.2 |

## Table S5: Socio-demographics variables for those harmed through lack of access compared to those harmed through treatment or care received (weighted analysis) (total n =988)

|  |  | Harmed through lack of access | | Harmed through treatment/ care received | | p |
| --- | --- | --- | --- | --- | --- | --- |
|  |  | n | % | n | % |  |
| **Demographics** | Groups |  |  |  |  |  |
| **Age (years)**  **(n=976)** | 18-24 | 48 | 13.3 | 77 | 12.5 | 0.37 |
|  | 25-34 | 56 | 15.5 | 118 | 19.2 |  |
|  | 35-44 | 61 | 16.9 | 113 | 18.4 |  |
|  | 45-54 | 62 | 17.1 | 114 | 18.6 |  |
|  | 55-64 | 58 | 16.0 | 90 | 14.7 |  |
|  | 65+ | 77 | 21.3 | 102 | 16.6 |  |
| **Sex**  **(n=958)** | Male | 138 | 39.1 | 214 | 35.4 | 0.25 |
|  | Female | 215 | 60.9 | 391 | 64.6 |  |
| **Ethnicity**  **(n=978)** | White British | 297 | 83.0 | 476 | 78.4 | **0.016** |
|  | White Other | 25 | 7.0 | 44 | 7.2 |  |
|  | Mixed | 16 | 4.5 | 15 | 2.5 |  |
|  | Asian | 12 | 3.4 | 34 | 5.6 |  |
|  | Black | 8 | 2.2 | 26 | 4.3 |  |
|  | Other | 0 | 0 | 12 | 2.0 |  |
| **Employment ^1^**  **(n=973)** | Full-time | 102 | 28.2 | 176 | 28.8 | 0.30 |
|  | Part-time employed | 54 | 14.9 | 68 | 11.1 |  |
|  | Self-employed | 34 | 9.4 | 64 | 10.5 |  |
|  | Economically inactive | 108 | 29.8 | 168 | 27.5 |  |
|  | Not working | 27 | 35.5 | 86 | 69.9 |  |
|  | Disability / long-term illness | 37 | 30.1 | 86 | 69.9 |  |

## Table S5 (continued): Socio-demographics variables for those harmed through lack of access compared to those harmed through treatment or care received (weighted analysis) (total n =988)

|  |  | Harmed through lack of access | | Harmed through treatment/ care received | | p |
| --- | --- | --- | --- | --- | --- | --- |
|  |  | n | % | n | % |  |
| **Region**  **(n=978)** | East Midlands | 17 | 4.7 | 50 | 8.1 | **0.034** |
|  | Eastern | 31 | 8.5 | 49 | 8.0 |  |
|  | London | 56 | 15.4 | 94 | 15.3 |  |
|  | North East | 9 | 2.5 | 31 | 5.0 |  |
|  | North West | 34 | 9.4 | 70 | 11.4 |  |
|  | Scotland | 34 | 9.4 | 42 | 6.8 |  |
|  | South East | 56 | 15.4 | 105 | 17.1 |  |
|  | South West | 37 | 10.2 | 42 | 6.8 |  |
|  | Wales | 32 | 8.8 | 31 | 5.0 |  |
|  | West Midlands | 31 | 8.5 | 60 | 9.8 |  |
|  | Yorkshire and Humberside | 26 | 7.2 | 41 | 6.7 |  |
| **Education** | GCSE/O-level | 43 | 11.9 | 93 | 15.2 | **0.004** |
|  | Vocational | 22 | 6.1 | 41 | 6.7 |  |
|  | A-level or equivalent | 68 | 18.8 | 160 | 26.1 |  |
|  | University degree or equivalent | 115 | 31.9 | 136 | 22.2 |  |
|  | Postgraduate degree or equivalent | 33 | 9.1 | 73 | 11.9 |  |
|  | Other | 30 | 8.3 | 37 | 6.0 |  |
|  | No formal qualifications | 50 | 13.9 | 72 | 11.8 |  |
| **Social grade ^2^** | A | 22 | 36.7 | 38 | 63.3 | 0.72 |
|  | B | 7 | 25.9 | 20 | 74.1 |  |
|  | C1 | 62 | 36.5 | 108 | 63.5 |  |
|  | C2 | 88 | 37.3 | 148 | 62.7 |  |
|  | D | 67 | 36.0 | 119 | 64.0 |  |
|  | E | 61 | 42.7 | 82 | 57.3 |  |
|  | Unknown | 54 | 35.3 | 99 | 64.7 |  |

^1^ Full-time – employed 30+ hours; Part-time – employed 29 hours or less; Economically inactive – full time students, still at school, retired or not in paid work due to long-term illness or disability; Not working – Unemployed and seeking work, Not in paid work for other reasons, or not working – housewife.

^2^ Social grade: National Readership Survey Classification A (highest grade) to E (lowest grade) <https://nrs.co.uk/nrs-print/lifestyle-and-classification-data/social-grade/>

## Table S6: Differences in total impact by socio-demographics and harm through access to treatment or care versus lack of access to treatment or care (* only significant results are reported for relevant post-hoc analyses)

|  |  | **No/mild** | | **Moderate** | | **Severe** | |  |  |
| --- | --- | --- | --- | --- | --- | --- | --- | --- | --- |
|  |  | **N** | **%** | **N** | **%** | **N** | **%** | **p** | **Post hoc analysis *** |
| **Age** | *18-24* | 21 | 17.1 | 55 | 44.7 | 47 | 38.2 | 0.49 | -- |
|  | *25-34* | 36 | 20.5 | 68 | 38.6 | 72 | 40.9 |  |  |
|  | *35-44* | 27 | 16.0 | 65 | 38.5 | 77 | 45.6 |  |  |
|  | *45-54* | 26 | 15.5 | 63 | 37.5 | 79 | 47.0 |  |  |
|  | *55-64* | 24 | 16.8 | 44 | 30.8 | 75 | 52.4 |  |  |
|  | *65+* | 34 | 19.5 | 63 | 36.2 | 77 | 44.3 |  |  |
| **Sex** | *Male* | 67 | 19.6 | 127 | 37.2 | 147 | 43.1 | 0.53 | N/A |
|  | *Female* | 99 | 16.7 | 226 | 38.2 | 353 | 37.8 |  |  |
| **Ethnicity** | *White British* | 130 | 17.2 | 276 | 36.6 | 348 | 46.2 | 0.74 | -- |
|  | *White Other* | 16 | 23.9 | 26 | 38.8 | 25 | 37.3 |  |  |
|  | *Mixed* | 4 | 13.3 | 14 | 46.7 | 12 | 40.0 |  |  |
|  | *Asian* | 9 | 19.6 | 20 | 43.5 | 17 | 37.0 |  |  |
|  | *Black* | 5 | 14.7 | 14 | 41.2 | 15 | 44.1 |  |  |
|  | *Other* | 2 | 20.0 | 2 | 20.0 | 6 | 60.0 |  |  |
| **Employment ^1^** | *Employed* | 57 | 21.2 | 120 | 44.6 | 92 | 34.2 | **<0.001** | No/ mild impact: Employed, self-employed, economically inactive vs. disability/ long-term illness;  Moderate impact: Employed, employed part-time; self-employed and economically inactive vs. disability/long-term illness;  High impact: Employed vs not working, and employed vs disability/ long-term illness. |
|  | *Employed*  *part-time* | 18 | 15.4 | 52 | 44.4 | 47 | 40.2 |  |  |
|  | *Self-employed* | 22 | 22.4 | 37 | 37.8 | 39 | 39.8 |  |  |
|  | *Economically*  *inactive* | 52 | 19.5 | 98 | 36.8 | 116 | 43.6 |  |  |
|  | *Not working* | 11 | 14.1 | 25 | 32.1 | 42 | 53.8 |  |  |
|  | *Disability /*  *long-term*  *illness* | 7 | 5.8 | 23 | 19.2 | 90 | 75.0 |  |  |
| **Region** | *East Midlands* | 10 | 15.4 | 21 | 32.3 | 34 | 52.3 | 0.42 | -- |
|  | *Eastern* | 22 | 29.3 | 25 | 33.3 | 28 | 37.3 |  |  |
|  | *London* | 23 | 15.6 | 65 | 44.2 | 59 | 40.1 |  |  |
|  | *North East* | 7 | 18.9 | 16 | 43.2 | 14 | 37.8 |  |  |
|  | *North West* | 15 | 14.7 | 40 | 39.2 | 47 | 46.1 |  |  |
|  | *Scotland* | 10 | 13.2 | 27 | 35.5 | 39 | 51.3 |  |  |
|  | *South East* | 28 | 17.5 | 59 | 36.9 | 73 | 45.6 |  |  |
|  | *South West* | 14 | 17.9 | 26 | 33.3 | 38 | 48.7 |  |  |
|  | *Wales* | 9 | 15.0 | 17 | 28.3 | 34 | 56.7 |  |  |
|  | *West Midlands* | 18 | 20.9 | 35 | 40.7 | 33 | 38.4 |  |  |
|  | *Yorkshire & Humberside* | 12 | 18.2 | 27 | 40.9 | 27 | 40.9 |  |  |

## Table S6 (continued): Differences in total impact by socio-demographics and harm through access to treatment/care versus lack of access to treatment or care (* only significant results are reported for relevant post-hoc analyses)

|  |  | **No/mild** | | **Moderate** | | **Severe** | |  |  |
| --- | --- | --- | --- | --- | --- | --- | --- | --- | --- |
|  |  | **N** | **%** | **N** | **%** | **N** | **%** | **p** | **Post hoc analysis *** |
| **Education** | GCSE/O-Level | 24 | 18.2 | 53 | 40.2 | 55 | 41.7 | **<0.001** | No/mild: A-level vs Postgraduate degree  Moderate: University degree vs No formal education  Severe: University degree and Postgraduate degree vs No formal education |
|  | Vocational | 8 | 13.1 | 22 | 36.1 | 31 | 50.8 |  |  |
|  | A-level or equivalent | 30 | 13.5 | 87 | 39.0 | 106 | 47.5 |  |  |
|  | University degree or equivalent | 42 | 17.3 | 111 | 45.7 | 90 | 37.0 |  |  |
|  | Postgraduate degree or equivalent | 30 | 29.1 | 35 | 34.0 | 38 | 36.9 |  |  |
|  | Other | 10 | 15.4 | 19 | 29.2 | 36 | 55.4 |  |  |
|  | No formal education | 20 | 17.1 | 29 | 24.8 | 68 | 58.1 |  |  |
| **Social grade ^2^** | *A* | 4 | 14.8 | 11 | 40.7 | 12 | 44.4 | **0.005** | Severe impact:  A vs B, D, E, Unknown vs B, D, E |
|  | *B* | 32 | 19.4 | 75 | 45.5 | 58 | 35.2 |  |  |
|  | *C1* | 39 | 17.0 | 96 | 41.9 | 94 | 41.0 |  |  |
|  | *C2* | 40 | 22.3 | 63 | 35.2 | 76 | 42.5 |  |  |
|  | *D* | 16 | 11.6 | 48 | 34.8 | 74 | 53.6 |  |  |
|  | *E* | 19 | 12.5 | 49 | 53.6 | 84 | 55.3 |  |  |
|  | *Unknown* | 16 | 26.2 | 17 | 27.9 | 28 | 44.8 |  |  |
| **Harmed through treatment or care vs lack of access** | Treatment or care | 120 | 20.1 | 216 | 36.2 | 260 | 43.6 | **0.019** | N/A |
|  | Lack of access | 45 | 13.0 | 141 | 40.6 | 161 | 46.4 |  |  |

^1^ Full-time – employed 30+ hours; Part-time – employed 29 hours or less; Economically inactive – full time students, still at school, retired or not in paid work due to long-term illness or disability; Not working – Unemployed and seeking work, Not in paid work for other reasons, or not working – housewife.

^2^ Social grade: National Readership Survey Classification A (highest grade) to E (lowest grade) <https://nrs.co.uk/nrs-print/lifestyle-and-classification-data/social-grade/>

Table S7. Proportion of participants reporting responses and differences by socio-demographic variables and access to treatment (weighted) (* Post-hoc analyses are shown only for significant pairwise comparisons, p<0.05)

|  | **Sharing experiences** | | | | **Seeking professional advice and support** | | | | **Making a formal complaint or taking legal action** | | | |
| --- | --- | --- | --- | --- | --- | --- | --- | --- | --- | --- | --- | --- |
| **Sex** | **n** | **%** | **p** | **Post-hoc*** | **n** | **%** | **p** | **Post-hoc*** | **n** | **%** | **p** | **Post-hoc*** |
| Male | 220 | 61.8 | <0.001 | -- | 212 | 59.7 | 0.63 | -- | 70 | 19.7 | 0.94 | **--** |
| Female | 450 | 73.3 |  |  | 357 | 58.1 |  |  | 122 | 19.9 |  |  |
| **Age** | **n** | **%** | **p** | **Post-hoc*** | **n** | **%** | **p** | **Post-hoc*** | **n** | **%** | **p** | **Post-hoc*** |
| 18-24 | 91 | 72.2 | 0.009 | 35-44 v 55-64 | 75 | 60.0 | 0.67 | -- | 27 | 21.4 | <0.001 | 25-34 v 65+  35-44 v 55-64  35-44 v 65+ |
| 25-34 | 131 | 73.2 |  |  | 100 | 56.2 |  |  | 41 | 23.0 |  |  |
| 35-44 | 131 | 74.9 |  |  | 108 | 61.7 |  |  | 50 | 28.6 |  |  |
| 45-54 | 129 | 72.5 |  |  | 101 | 56.7 |  |  | 35 | 19.7 |  |  |
| 55-64 | 90 | 59.6 |  |  | 95 | 62.9 |  |  | 22 | 14.6 |  |  |
| 65+ | 113 | 62.8 |  |  | 100 | 55.9 |  |  | 20 | 11.1 |  |  |
| **Social grade ^1^** | **n** | **%** | **p** | **Post-hoc*** | **n** | **%** | **p** | **Post-hoc*** | **n** | **%** | **p** | **Post-hoc*** |
| A | 24 | 88.9 | 0.003 | *--* | 12 | 44.4 | 0.44 | -- | 3 | 10.7 | 0.31 | -- |
| B | 122 | 70.9 |  |  | 106 | 61.6 |  |  | 39 | 22.8 |  |  |
| C1 | 176 | 74.3 |  |  | 145 | 60.9 |  |  | 51 | 21.4 |  |  |
| C2 | 140 | 73.3 |  |  | 103 | 53.9 |  |  | 28 | 14.7 |  |  |
| D | 86 | 59.7 |  |  | 82 | 57.3 |  |  | 26 | 18.2 |  |  |
| E | 96 | 62.3 |  |  | 93 | 60.4 |  |  | 35 | 22.7 |  |  |
| Unknown | 40 | 64.5 |  |  | 39 | 62.9 |  |  | 13 | 21.0 |  |  |

## Table S7 (continued) Proportion of participants reporting responses and differences by socio-demographic variables and access to treatment (weighted) (* Post-hoc analyses are shown only for significant pairwise comparisons, p<0.05)

|  | **Sharing experiences** | | | | **Seeking professional advice and support** | | | | **Making a formal complaint or taking legal action** | | | |
| --- | --- | --- | --- | --- | --- | --- | --- | --- | --- | --- | --- | --- |
| **Employment** | **n** | **%** | **p** | **Post-hoc*** | **n** | **%** | **p** | **Post-hoc*** | **n** | **%** | **p** | **Post-hoc*** |
| Employed full-time | 206 | 73.3 | 0.11 |  | 156 | 55.5 | 0.020 | -- | 64 | 22.9 | 0.001 | Employed PT v long term illness  Economically inactive v long term illness |
| Employed part-time | 83 | 67.5 |  |  | 61 | 49.6 |  |  | 15 | 12.2 |  |  |
| Self-employed | 69 | 69.7 |  |  | 62 | 62.6 |  |  | 18 | 18.2 |  |  |
| Economically inactive | 186 | 67.1 |  |  | 162 | 58.7 |  |  | 44 | 15.9 |  |  |
| Not working: other reasons | 61 | 77.2 |  |  | 55 | 69.6 |  |  | 14 | 17.7 |  |  |
| Not working: disability / long-term illness | 76 | 61.3 |  |  | 83 | 66.9 |  |  | 39 | 31.5 |  |  |
| **Education** | **n** | **%** | **p** | **Post-hoc*** | **n** | **%** | **p** | **Post-hoc*** | **n** | **%** | **p** | **Post-hoc*** |
| GCSE / O-Level | 92 | 67.2 | 0.038 | Bachelor’s v No formal qual | 75 | 55.1 | 0.12 |  | 25 | 18.2 | 0.009 |  |
| Vocational | 47 | 74.6 |  |  | 40 | 63.5 |  |  | 19 | 30.2 |  |  |
| A level or equivalent | 165 | 71.7 |  |  | 140 | 60.9 |  |  | 35 | 15.2 |  |  |
| Undergraduate degree or equivalent | 184 | 72.2 |  |  | 164 | 64.6 |  |  | 58 | 22.8 |  |  |
| Postgraduate degree or equivalent | 76 | 71.0 |  |  | 56 | 52.3 |  |  | 30 | 28.0 |  |  |
| Other | 48 | 70.6 |  |  | 39 | 56.5 |  |  | 11 | 16.2 |  |  |
| No formal qualifications | 70 | 56.0 |  |  | 64 | 51.2 |  |  | 17 | 13.6 |  |  |

## Table S7 (continued). Proportion of participants reporting responses and differences by socio-demographic variables and access to treatment (weighted) (* Post-hoc analyses are shown only for significant pairwise comparisons, p<0.05)

|  | **Sharing experiences** | | | | **Seeking professional advice and support** | | | | **Making a formal complaint or taking legal action** | | | |
| --- | --- | --- | --- | --- | --- | --- | --- | --- | --- | --- | --- | --- |
| **Geographical region** | **n** | **%** | **p** | **Post-hoc*** | **n** | **%** | **p** | **Post-hoc*** | **n** | **%** | **p** | **Post-hoc*** |
| East Midlands | 43 | 64.2 | 0.79 |  | 45 | 66.2 | 0.82 |  | 19 | 27.9 | 0.029 | North East v North West; |
| Eastern | 60 | 75.0 |  |  | 45 | 56.3 |  |  | 13 | 16.3 |  |  |
| London | 102 | 67.1 |  |  | 87 | 57.2 |  |  | 35 | 22.9 |  |  |
| North East | 27 | 67.5 |  |  | 23 | 57.5 |  |  | 15 | 37.5 |  |  |
| North West | 76 | 71.0 |  |  | 69 | 63.9 |  |  | 12 | 11.2 |  |  |
| Scotland | 50 | 65.8 |  |  | 48 | 62.3 |  |  | 19 | 25.0 |  |  |
| South East | 112 | 69.6 |  |  | 91 | 56.5 |  |  | 32 | 19.9 |  |  |
| South West | 51 | 64.6 |  |  | 49 | 61.3 |  |  | 15 | 18.8 |  |  |
| Wales | 46 | 73.0 |  |  | 32 | 50.8 |  |  | 11 | 17.5 |  |  |
| West Midlands | 64 | 68.1 |  |  | 53 | 56.4 |  |  | 15 | 16.0 |  |  |
| Yorkshire and Humber | 51 | 77.3 |  |  | 39 | 58.2 |  |  | 11 | 16.4 |  |  |
| **Total Impact** | **n** | **%** | **p** | **Post-hoc*** | **n** | **%** | **p** | **Post-hoc*** | **n** | **%** | **p** | **Post-hoc*** |
| No/mild | 122 | 73.5 | 0.37 |  | 67 | 41.1 | <0.001 | No/mild v moderate, no/mild v severe, and moderate v severe | 21 | 12.7 | 0.002 | No/mild v severe |
| Moderate | 251 | 70.1 |  |  | 198 | 55.3 |  |  | 66 | 18.4 |  |  |
| Severe | 289 | 67.7 |  |  | 297 | 69.7 |  |  | 106 | 24.8 |  |  |
| **Access / lack of access** | **n** | **%** | **p** | **Post-hoc*** | **n** | **%** | **p** | **Post-hoc*** | **n** | **%** | **p** | **Post-hoc*** |
| Lack of access | 246 | 68.0 | 0.315 |  | 235 | 64.9 | 0.006 |  | 44 | 12.2 | <0.001 |  |
| Treatment or care | 436 | 71.0 |  |  | 344 | 56.0 |  |  | 149 | 24.3 |  |  |

**^1^**Social grade: National Readership Survey Classification A (highest grade) to E (lowest grade) <https://nrs.co.uk/nrs-print/lifestyle-and-classification-data/social-grade/>

## Table S8a: Associations between Sharing experiences and participant characteristics, estimated using logistic regression (n=920)

|  | **N** | **Shared experiences (%)** | **Odds ratio** | **95% CI** | **p** |
| --- | --- | --- | --- | --- | --- |
| **Sex** |  |  |  |  |  |
| Male (reference category) | 220 | 61.8 | 1 |  |  |
| Female | 450 | 73.3 | 1.63 | 1.20 to 2.22 | **0.002** |
| **Age** |  |  |  |  |  |
| 18-24 (reference category) | 91 | 72.2 | 1 |  | 0.061 |
| 25-34 | 131 | 73.2 | 1.04 | 0.58 to 1.88 | 0.89 |
| 35-44 | 131 | 74.9 | 1.23 | 0.68 to 2.24 | 0.50 |
| 45-54 | 129 | 72.5 | 1.07 | 0.59 to 1.94 | 0.83 |
| 55-64 | 90 | 59.6 | 0.57 | 0.32 to 1.02 | **0.06** |
| 65+ | 113 | 62.8 | 0.71 | 0.38 to 1.33 | 0.29 |
| **Employment ^1^** |  |  |  |  |  |
| Full-time (reference category) | 206 | 73.3 | 1 |  | 0.29 |
| Part-time | 83 | 67.5 | 0.99 | 0.58 to 1.68 | 0.96 |
| Self-employed | 69 | 69.7 | 0.85 | 0.49 to 1.48 | 0.56 |
| Economically inactive | 186 | 67.1 | 1.07 | 0.63 to 1.81 | 0.81 |
| Not working | 61 | 77.2 | 1.63 | 0.81 to 3.30 | 0.17 |
| Disability/ long-term illness | 76 | 61.3 | 0.69 | 0.40 to 1.22 | 0.20 |
| **Education** |  |  |  |  |  |
| GSCE (reference category) | 92 | 67.2 | 1 |  | 0.77 |
| Vocational | 47 | 74.6 | 1.17 | 0.57 to 2.40 | 0.67 |
| A-level or equivalent | 165 | 71.7 | 1.02 | 0.62 to 1.69 | 0.93 |
| Undergraduate degree or equivalent | 184 | 72.2 | 1.15 | 0.69 to 1.90 | 0.60 |
| Postgraduate degree or equivalent | 76 | 71.0 | 0.88 | 0.46 to 1.66 | 0.69 |
| Other | 48 | 70.6 | 0.95 | 0.47 to 1.91 | 0.88 |
| No formal education | 70 | 56.0 | 0.74 | 0.42 to 1.32 | 0.31 |
| **Social grade 2** |  |  |  |  |  |
| A (reference category) | 24 | 88.9 | 1 |  | **0.017** |
| B | 122 | 70.9 | 5.60 | 1.22 to 25.69 | **0.027** |
| C1 | 176 | 74.3 | 1.41 | 0.71 to 2.80 | 0.33 |
| C2 | 140 | 73.3 | 1.52 | 0.79 to 2.94 | 0.21 |
| D | 86 | 59.7 | 1.62 | 0.82 to 3.22 | 0.17 |
| E | 96 | 62.3 | 0.77 | 0.38 to 1.54 | 0.46 |
| Unknown | 40 | 64.5 | 0.99 | 0.50 to 1.96 | 0.97 |

## Table S8a (continued): Associations between Sharing experiences and participant characteristics, estimated using logistic regression (n=920)

|  | **N** | **Shared experiences (%)** | **Odds ratio** | **95% CI** | **p** |
| --- | --- | --- | --- | --- | --- |
| **Region** |  |  |  |  |  |
| East Midlands (reference category) | 43 | 64.2 | 1 |  | 0.90 |
| Eastern | 60 | 75.0 | 1.47 | 0.68 to 3.19 | 0.33 |
| London | 102 | 67.1 | 1.19 | 0.61 to 2.31 | 0.61 |
| North East | 27 | 67.5 | 0.97 | 0.40 to 2.38 | 0.95 |
| North West | 76 | 71.0 | 1.53 | 0.75 to 3.13 | 0.25 |
| Scotland | 50 | 65.8 | 1.22 | 0.58 to 2.56 | 0.61 |
| South East | 112 | 69.6 | 1.28 | 0.67 to 2.46 | 0.45 |
| South West | 51 | 64.6 | 1.06 | 0.50 to 2.24 | 0.88 |
| Wales | 46 | 73.0 | 1.95 | 0.85 to 4.52 | 0.12 |
| West Midlands | 64 | 68.1 | 1.27 | 0.61 to 2.63 | 0.52 |
| Yorkshire and Humber | 51 | 77.3 | 1.51 | 0.67 to 3.39 | 0.32 |
| **Total Impact** |  |  |  |  |  |
| No/mild (reference category) | 122 | 73.5 | 1 |  | 0.65 |
| Moderate | 251 | 70.1 | 0.81 | 0.52 to 1.26 | 0.35 |
| Severe | 289 | 67.7 | 0.86 | 0.56 to 1.34 | 0.51 |
| **Harmed through access** |  |  |  |  |  |
| Harmed through lack of access to care (reference category) | 246 | 68.0 | 1 |  | 0.68 |
| Harmed by NHS treatment or care | 436 | 71.0 | 1.07 | 0.78 to 1.46 |  |

^1^ Full-time – employed 30+ hours; Part-time – employed 29 hours or less; Economically inactive – full time students, still at school, retired or not in paid work due to long-term illness or disability; Not working – Unemployed and seeking work, Not in paid work for other reasons, or not working – housewife.

^2^ Social grade: National Readership Survey Classification A (highest grade) to E (lowest grade) <https://nrs.co.uk/nrs-print/lifestyle-and-classification-data/social-grade/>

## Table S8b: Associations between Seeking professional support and advice and participant characteristics, estimated using logistic regression (n=887)

|  | **N** | **Seeking professional support (%)** | **Odds ratio** | **95% CI** | **p** |
| --- | --- | --- | --- | --- | --- |
| **Sex** |  |  |  |  |  |
| Male (reference category) | 212 | 59.7 | 1 |  |  |
| Female | 357 | 58.1 | 0.89 | 0.66 to 1.21 | 0.46 |
| **Age** |  |  |  |  |  |
| 18-24 | 75 | 60.0 |  |  | 0.75 |
| 25-34 | 100 | 56.2 | 0.66 | 0.38 to 1.14 | 0.14 |
| 35-44 | 108 | 61.7 | 0.77 | 0.44 to 1.34 | 0.35 |
| 45-54 | 101 | 56.7 | 0.81 | 0.46 to 1.41 | 0.45 |
| 55-64 | 95 | 62.9 | 0.85 | 0.48 to 1.49 | 0.56 |
| 65+ | 100 | 55.9 | 0.73 | 0.40 to 1.33 | 0.30 |
| **Employment ^1^** |  |  |  |  |  |
| Full-time (reference category) | 156 | 55.5 | 1 |  | 0.064 |
| Part-time | 61 | 49.6 | 0.82 | 0.50 to 1.33 | 0.41 |
| Self-employed | 62 | 62.6 | 1.47 | 0.86 to 2.51 | 0.16 |
| Economically inactive | 162 | 58.7 | 1.24 | 0.75 to 2.04 | 0.40 |
| Not working | 55 | 69.6 | 2.27 | 1.20 to 4.32 | **0.012** |
| Disability/ long-term illness | 83 | 66.9 | 1.24 | 0.72 to 2.16 | 0.44 |
| **Social grade ^2^** |  |  |  |  |  |
| A (reference category) | 12 | 44.4 | 1 |  | 0.310 |
| B | 106 | 61.6 | 0.40 | 0.14 to 1.15 | **0.09** |
| C1 | 145 | 60.9 | 1.02 | 0.51 t 2.05 | 0.95 |
| C2 | 103 | 53.9 | 0.79 | 0.40 to 1.54 | 0.49 |
| D | 82 | 57.3 | 0.66 | 0.33 to 1.32 | 0.24 |
| E | 93 | 60.4 | 0.68 | 0.33 to 1.40 | 0.30 |
| Unknown | 39 | 62.9 | 0.63 | 0.31 to 1.28 | 0.20 |
| **Education** |  |  |  |  |  |
| GSCE (reference category) | 75 | 55.1 | 1 |  | 0.063 |
| Vocational | 40 | 63.5 | 1.47 | 0.74 to 2.91 | 0.27 |
| A-level or equivalent | 140 | 60.9 | 1.22 | 0.76 to 1.97 | 0.41 |
| Undergraduate degree or equivalent | 164 | 64.6 | 1.72 | 1.06 to 2.78 | **0.028** |
| Postgraduate degree or equivalent | 56 | 52.3 | 1.08 | 0.59 to 1.97 | 0.80 |
| Other | 39 | 56.5 | 0.91 | 0.47 to 1.76 | 0.78 |
| No formal education | 64 | 51.2 | 0.77 | 0.44 to 1.36 | 0.37 |

## Table S8b (continued): Associations between Seeking professional support and advice and participant characteristics, estimated using logistic regression (n=887)

|  | **N participants** | **Seeking professional support (%)** | **Odds ratio** | **95% CI** | **p** |
| --- | --- | --- | --- | --- | --- |
| **Geographical region** |  |  |  |  |  |
| East Midlands (reference category) | 45 | 66.2 | 1 |  | 0.68 |
| Eastern | 45 | 56.3 | 0.57 | 0.27 to 1.21 | 0.14 |
| London | 87 | 57.2 | 0.60 | 0.31 to 1.18 | 0.14 |
| North East | 23 | 57.5 | 0.68 | 0.28 to 1.67 | 0.40 |
| North West | 69 | 63.9 | 0.79 | 0.39 to 1.62 | 0.52 |
| Scotland | 48 | 62.3 | 0.71 | 0.33 to 1.53 | 0.39 |
| South East | 91 | 56.5 | 0.58 | 0.30 to 1.12 | 0.11 |
| South West | 49 | 61.3 | 0.56 | 0.26 to 1.20 | 0.14 |
| Wales | 32 | 50.8 | 0.40 | 0.18 to 0.88 | **0.022** |
| West Midlands | 53 | 56.4 | 0.61 | 0.30 to 1.26 | 0.18 |
| Yorkshire and Humber | 39 | 58.2 | 0.52 | 0.24 to 1.13 | 0.10 |
| **Impact** |  |  |  |  |  |
| No/mild (reference category) | 67 | 41.1 | 1 |  | **<0.001** |
| Moderate | 198 | 55.3 | 1.80 | 1.21 to 2.69 | **0.004** |
| Severe | 297 | 69.7 | 3.82 | 2.53 to 5.76 | **<0.001** |
| **Harmed through access** |  |  |  |  |  |
| Harmed through lack of access to care (reference category) | 235 | 64.9 | 1 |  |  |
| Harmed through NHS treatment or care | 344 | 56.0 | 0.67 | 0.50 to 0.91 | **0.01** |

^1^ Full-time – employed 30+ hours; Part-time – employed 29 hours or less; Economically inactive – full time students, still at school, retired or not in paid work due to long-term illness or disability; Not working – Unemployed and seeking work, Not in paid work for other reasons, or not working – housewife.

^2^ Social grade: National Readership Survey Classification A (highest grade) to E (lowest grade) <https://nrs.co.uk/nrs-print/lifestyle-and-classification-data/social-grade/>

## Table S8c: Associations between Formal action and participant characteristics, estimated using logistic regression (n=887)

|  | **N** | **Formal action (%)** | **Odds ratio** | **95% CI** | **p** |
| --- | --- | --- | --- | --- | --- |
| **Sex** |  |  |  |  |  |
| Male (reference category) | 70 | 19.7 | 1 |  |  |
| Female | 122 | 19.9 | 0.91 | 0.63 to 1.31 | 0.60 |
| **Age** |  |  |  |  |  |
| 18-24 (reference category) | 27 | 21.4 | 1 |  | **0.018** |
| 25-34 | 41 | 23.0 | 0.82 | 0.42 to 1.57 | 0.54 |
| 35-44 | 50 | 28.6 | 1.01 | 0.53 to 1.93 | 0.99 |
| 45-54 | 35 | 19.7 | 0.68 | 0.34 to 1.33 | 0.26 |
| 55-64 | 22 | 14.6 | 0.47 | 0.23 to 0.96 | **0.038** |
| 65+ | 20 | 11.1 | 0.31 | 0.14 to 0.69 | **0.004** |
| **Employment ^1^** |  |  |  |  |  |
| Full-time (reference category) | 64 | 22.9 | 1 |  | **0.09** |
| Part-time | 15 | 12.2 | 0.49 | 0.25 to 0.96 | **0.038** |
| Self-employed | 18 | 18.2 | 0.88 | 0.46 to 1.67 | 0.69 |
| Economically inactive | 44 | 15.9 | 1.08 | 0.59 to 1.98 | 0.79 |
| Not working | 14 | 17.7 | 0.72 | 0.33 to 1.57 | 0.41 |
| Disability/ long-term illness | 39 | 31.5 | 1.46 | 0.79 to 2.69 | 0.23 |
| **Social grade ^2^** |  |  |  |  |  |
| A (reference category) | 3 | 10.7 | 1 |  | 0.71 |
| B | 39 | 22.8 | 0.31 | 0.05 to 1.79 | 0.19 |
| C1 | 51 | 21.4 | 1.19 | 0.52 to 2.72 | 0.68 |
| C2 | 28 | 14.7 | 1.14 | 0.51 to 2.53 | 0.75 |
| D | 26 | 18.2 | 0.87 | 0.38 to 2.02 | 0.75 |
| E | 35 | 22.7 | 1.17 | 0.49 to 2.80 | 0.72 |
| Unknown | 13 | 21.0 | 1.07 | 0.46 to 2.48 | 0.88 |
| **Education** |  |  |  |  |  |
| GSCE (reference category) | 25 | 18.2 | 1 |  | **0.022** |
| Vocational | 19 | 30.2 | 2.36 | 1.08 to 5.13 | **0.031** |
| A-level or equivalent | 35 | 15.2 | 0.85 | 0.45 to 1.59 | 0.61 |
| Undergraduate degree or equivalent | 58 | 22.8 | 1.85 | 1.01 to 3.39 | **0.045** |
| Postgraduate degree or equivalent | 30 | 28.0 | 2.18 | 1.05 to 4.49 | **0.036** |
| Other | 11 | 16.2 | 1.09 | 0.46 to 2.58 | 0.85 |
| No formal education | 17 | 13.6 | 1.27 | 0.59 to 2.73 | 0.54 |

## Table S8c (continued): Associations between Formal action and participant characteristics, estimated using logistic regression (n=887)

|  | **N** | **Formal action (%)** | **Odds ratio** | **95% CI** | **p** |
| --- | --- | --- | --- | --- | --- |
| **Geographical region** |  |  |  |  |  |
| East Midlands (reference category) | 19 | 27.9 | 1 |  | 0.16 |
| Eastern | 13 | 16.3 | 0.58 | 0.23 to 1.43 | 0.24 |
| London | 35 | 22.9 | 0.87 | 0.42 to 1.82 | 0.71 |
| North East | 15 | 37.5 | 1.67 | 0.66 to 4.24 | 0.28 |
| North West | 12 | 11.2 | 0.37 | 0.15 to 0.89 | **0.03** |
| Scotland | 19 | 25.0 | 1.05 | 0.46 to 2.381 | 0.91 |
| South East | 32 | 19.9 | 0.73 | 0.35 to 1.50 | 0.39 |
| South West | 15 | 18.8 | 0.84 | 0.35 to 2.01 | 0.69 |
| Wales | 11 | 17.5 | 0.76 | 0.31 to 1.91 | 0.56 |
| West Midlands | 15 | 16.0 | 0.62 | 0.27 to 1.44 | 0.26 |
| Yorkshire and Humber | 11 | 16.4 | 0.52 | 0.20 to 1.33 | 0.17 |
| **Impact** |  |  |  |  |  |
| No/mild (reference category) | 21 | 12.7 | 1 |  | **0.002** |
| Moderate | 66 | 18.4 | 1.61 | 0.92 to 2.83 | **0.01** |
| Severe | 106 | 24.8 | 2.55 | 1.47 to 4.42 | **<0.001** |
| **Access** |  |  |  |  |  |
| Harmed through lack of access to care (reference category) | 44 | 12.2 | 1 |  |  |
| Harmed through NHS treatment or care | 149 | 24.3 | 2.47 | 1.66 to 3.69 | **<0.001** |

^1^ Full-time – employed 30+ hours; Part-time – employed 29 hours or less; Economically inactive – full time students, still at school, retired or not in paid work due to long-term illness or disability; Not working – Unemployed and seeking work, Not in paid work for other reasons, or not working – housewife.

^2^ Social grade: National Readership Survey Classification A (highest grade) to E (lowest grade) <https://nrs.co.uk/nrs-print/lifestyle-and-classification-data/social-grade/>

## Table S9. Helpfulness of PALS (for n=114 had used PALS) and handling of formal complaint (for n=168 who had made a formal complaint) (weighted)

|  | **Frequency (n)** | **Percent (%)** |
| --- | --- | --- |
| **Helpfulness of PALS** |  |  |
| Very helpful | 25 | 21.5 |
| Fairly helpful | 34 | 29.5 |
| Not very helpful | 24 | 21.2 |
| Not at all helpful | 28 | 24.6 |
| **Handling of formal complaint** |  |  |
| Very well | 17 | 10.2 |
| Fairly well | 33 | 19.8 |
| Not very well | 38 | 22.8 |
| Not at all well | 67 | 40.2 |

## Table S10. Main reason for not pursuing a legal claim for financial compensation (weighted) (n=962 for those who had not pursued a legal claim)

|  | **Frequency (n)** | **Percent (%)** |
| --- | --- | --- |
| Did not want to make a financial claim against the NHS | 208 | 21.6 |
| Did not want financial compensation | 175 | 18.2 |
| Did not occur to respondent | 132 | 13.7 |
| Too stressful or emotionally difficult | 91 | 9.4 |
| Thought it would be too time-consuming | 48 | 5.0 |
| Did not know how to make a claim | 46 | 4.8 |
| Thought it would be too costly | 27 | 2.8 |
| Too unwell | 25 | 2.6 |
| Solicitor/claims company advised against it | 15 | 1.5 |
| Other reason *(offered by respondent^1^)* | 122 | 12.7 |
| No reason | 58 | 6.1 |

^1^Other reasons given included: no point in making a claim; the harm was too mild, and nobody was to blame.

## Table S11a. Variations in responses desired (an apology, an explanation or emotional support) by the NHS following harm by sociodemographic factors and harm through access or lack of access to treatment or care (* post hoc analysis reported for significant results (p<0.05))

|  |  | **An apology** | | | | **An explanation** | | | | **Emotional support** | | | |
| --- | --- | --- | --- | --- | --- | --- | --- | --- | --- | --- | --- | --- | --- |
|  |  | **Yes** | | | | **Yes** | | | | **Yes** | | | |
|  |  | **n** | **%** | **p** | **Post-hoc*** | **n** | **%** | **p** | **Post-hoc*** | **n** | **%** | **p** | **Post-hoc*** |
|  | **Age** |  |  |  |  |  |  |  |  |  |  |  |  |
| **Age** | *18-24* | 33 | 26.2 | 0.65 |  | 43 | 34.1 | 0.37 |  | 39 | 31.0 | **<0.001** | 18-24 v 55-64  18-24 v 65+  25-34 v 65+  45-54 v 65+ |
|  | *25-34* | 57 | 31.8 |  |  | 63 | 35.2 |  |  | 45 | 25.3 |  |  |
|  | *35-44* | 56 | 32.0 |  |  | 64 | 36.6 |  |  | 42 | 24.0 |  |  |
|  | *45-54* | 51 | 28.7 |  |  | 72 | 40.4 |  |  | 45 | 25.3 |  |  |
|  | *55-64* | 36 | 23.8 |  |  | 49 | 32.5 |  |  | 24 | 15.9 |  |  |
|  | *65+* | 35 | 19.6 |  |  | 53 | 29.4 |  |  | 22 | 12.2 |  |  |
|  | *Sex* |  |  |  |  |  |  |  |  |  |  |  |  |
| **Sex** | *Male* | 74 | 20.8 | **<0.001** | N/A | 106 | 29.9 | **0.017** | N/A | 58 | 16.3 | **0.002** | N/A |
|  | *Female* | 188 | 30.6 |  |  | 230 | 37.4 |  |  | 152 | 24.8 |  |  |
|  | *Employment ^2^* |  |  |  |  |  |  |  |  |  |  |  |  |
| **Employment ^2^** | *Employed full-time* | 76 | 27.1 | 0.08 |  | 95 | 33.8 | **<0.001** | PT v not working;  PT v disability  Econ inactive v not working;  Econ inactive v disability | 56 | 20.0 | **<0.001** |  |
|  | *Employed part-time* | 35 | 28.5 |  |  | 35 | 28.2 |  |  | 35 | 28.5 |  |  |
|  | *Self-employed* | 23 | 23.2 |  |  | 36 | 36.4 |  |  | 17 | 17.2 |  |  |
|  | *Economically inactive* | 62 | 22.5 |  |  | 80 | 28.9 |  |  | 42 | 15.2 |  |  |
|  | *Not working* | 25 | 31.6 |  |  | 39 | 49.4 |  |  | 25 | 31.6 |  |  |
|  | *Disability / long-term illness* | 45 | 36.3 |  |  | 57 | 46.3 |  |  | 41 | 33.1 |  |  |

## Table S11a (continued). Variations in responses desired (an apology, an explanation or emotional support) by the NHS following harm by sociodemographic factors and harm through access or lack of access to treatment or care (* post hoc analysis reported for significant results (p<0.05))

|  | **An apology** | | | | **An explanation** | | | | **Emotional support** | | | |
| --- | --- | --- | --- | --- | --- | --- | --- | --- | --- | --- | --- | --- |
|  | **Yes** | | | | **Yes** | | | | **Yes** | | | |
|  | **n** | **%** | **p** | **Post-hoc*** | **n** | **%** | **p** | **Post-hoc*** | **n** | **%** | **p** | **Post-hoc*** |
| **Education** |  |  |  |  |  |  |  |  |  |  |  |  |
| *GCSE / O-Level* | 31 | 22.8 | 0.52 | -- | 46 | 33.6 | **0.003** | A-level v no qualifications | 22 | 16.2 | **<0.001** | GCSE v A-level;  A-level v Postgraduate |
| *Vocational* | 20 | 31.7 |  |  | 27 | 42.9 |  |  | 17 | 27.0 |  |  |
| *A level or equivalent* | 70 | 30.4 |  |  | 102 | 44.3 |  |  | 74 | 32.0 |  |  |
| *Bachelor’s degree or equivalent* | 72 | 28.3 |  |  | 86 | 33.9 |  |  | 54 | 21.3 |  |  |
| *Postgraduate degree or equivalent* | 29 | 27.1 |  |  | 33 | 30.6 |  |  | 15 | 14.0 |  |  |
| *Other* | 15 | 22.1 |  |  | 19 | 27.9 |  |  | 11 | 16.2 |  |  |
| *No formal qualifications* | 30 | 24.0 |  |  | 30 | 24.0 |  |  | 23 | 18.4 |  |  |
| **Social grade ^1^** |  |  |  |  |  |  |  |  |  |  |  |  |
| *A* | 5 | 18.5 | 0.20 | -- | 8 | 28.6 | **0.003** | B v D  C2 v D  D v E | 4 | 14.8 | 0.13 | **--** |
| *B* | 57 | 33.1 |  |  | 69 | 40.1 |  |  | 29 | 17.0 |  |  |
| *C1* | 68 | 28.7 |  |  | 82 | 34.5 |  |  | 59 | 24.8 |  |  |
| *C2* | 43 | 22.5 |  |  | 78 | 40.8 |  |  | 35 | 18.3 |  |  |
| *D* | 35 | 24.3 |  |  | 30 | 20.8 |  |  | 39 | 27.3 |  |  |
| *E* | 39 | 25.3 |  |  | 57 | 36.8 |  |  | 36 | 23.4 |  |  |
| **Access / lack of access** |  |  |  |  |  |  |  |  |  |  |  |  |
| *Harmed through lack of access* | 60 | 16.6 | **<0.001** | N/A | 103 | 28.5 | **<0.001** | N/A | 73 | 20.1 | 0.25 | **--** |
| *Harmed by treatment or care* | 206 | 33.6 |  |  | 239 | 38.9 |  |  | 143 | 23.3 |  |  |

^1^ Social grade: National Readership Survey Classification A (highest grade) to E (lowest grade) <https://nrs.co.uk/nrs-print/lifestyle-and-classification-data/social-grade/>

^2^ Full-time – employed 30+ hours; Part-time – employed 29 hours or less; Economically inactive – full time students, still at school, retired or not in paid work due to long-term illness or disability; Not working – Unemployed and seeking work, Not in paid work for other reasons, or not working – housewife.

## Table S11b. Variations in responses desired (treatment/care to redress harm; access to treatment previously refused, and staff training) by the NHS following harm by sociodemographic factors and harm through access or lack of access to treatment or care (* post hoc analysis reported for significant results (p<0.05))

|  | **Treatment/care to redress harm** | | | | **Access to treatment previously refused** | | | | **Staff training** | | | |
| --- | --- | --- | --- | --- | --- | --- | --- | --- | --- | --- | --- | --- |
|  | **Yes** | | | | **Yes** | | | | **Yes** | | | |
|  | **n** | **%** | **p** | **Post-hoc*** | **n** | **%** | **p** | **Post-hoc*** | **n** | **%** | **p** | **Post-hoc*** |
| **Age** |  |  |  |  |  |  |  |  |  |  |  |  |
| 18-24 | 57 | 45.2 | **0.002** | 35-44 v 45-54  45-54 v 65+ | 44 | 34.9 | **0.009** | None signif | 30 | 23.8 | **0.018** | 25-34 v 65+ |
| 25-34 | 90 | 50.3 |  |  | 60 | 33.7 |  |  | 54 | 30.3 |  |  |
| 35-44 | 62 | 35.4 |  |  | 61 | 34.9 |  |  | 52 | 29.7 |  |  |
| 45-54 | 97 | 54.5 |  |  | 55 | 30.9 |  |  | 49 | 27.5 |  |  |
| 55-64 | 66 | 43.7 |  |  | 33 | 21.9 |  |  | 31 | 20.5 |  |  |
| 65+ | 67 | 37.2 |  |  | 39 | 21.7 |  |  | 30 | 16.8 |  |  |
| **Sex** |  |  |  |  |  |  |  |  |  |  |  |  |
| Male | 164 | 46.2 | 0.35 |  | 107 | 30.1 | 0.58 |  | 69 | 19.4 | **0.003** |  |
| Female | 265 | 43.1 |  |  | 175 | 28.5 |  |  | 173 | 28.1 |  |  |
| **Social grade ^1^** |  |  |  |  |  |  |  |  |  |  |  |  |
| A | 11 | 39.3 | 0.61 |  | 10 | 35.7 | 0.54 |  | 0 | 0 | **<0.001** | *A v B*  *A v C1*  *A v C2*  *A v E*  *B v D*  *C1 v D; D v E* |
| B | 86 | 50.0 |  |  | 54 | 31.4 |  |  | 53 | 30.8 |  |  |
| C1 | 103 | 43.5 |  |  | 69 | 29.0 |  |  | 64 | 27.0 |  |  |
| C2 | 79 | 41.1 |  |  | 58 | 30.4 |  |  | 51 | 26.7 |  |  |
| D | 65 | 45.5 |  |  | 35 | 24.5 |  |  | 20 | 13.9 |  |  |
| E | 66 | 42.9 |  |  | 53 | 34.2 |  |  | 44 | 28.6 |  |  |
| **Access / lack of access** |  |  |  |  |  |  |  |  |  |  |  |  |
| Harmed through lack of access | 194 | 53.6 | **<0.001** |  | 141 | 39.0 | **<0.001** |  | 68 | 18.8 | **<0.001** |  |
| Harmed by treatment or care | 243 | 39.6 |  |  | 151 | 24.6 |  |  | 177 | 28.8 |  |  |

^1^ Social grade: National Readership Survey Classification A (highest grade) to E (lowest grade) <https://nrs.co.uk/nrs-print/lifestyle-and-classification-data/social-grade/>

## Table S11 (continued Variations in responses desired (treatment/care to redress harm; access to treatment previously refused, and staff training) by the NHS following harm by sociodemographic factors and harm through access or lack of access to treatment or (* post hoc analysis reported for significant results (p<0.05))

|  | **Treatment/care to redress harm** | | | | **Access to treatment previously refused** | | | | **Staff training** | | | |
| --- | --- | --- | --- | --- | --- | --- | --- | --- | --- | --- | --- | --- |
|  | **Yes** | | | | **Yes** | | | | **Yes** | | | |
|  | **n** | **%** | **p** | **Post-hoc*** | **n** | **%** | **p** | **Post-hoc*** | **n** | **%** | **p** | **Post-hoc*** |
| **Employment ^2^** |  |  |  |  |  |  |  |  |  |  |  |  |
| Employed full-time | 125 | 44.6 | 0.09 |  | 82 | 29.2 | **0.025** | Econ inactive v not working | 79 | 28.2 | 0.10 |  |
| Employed part-time | 61 | 49.6 |  |  | 35 | 28.5 |  |  | 25 | 20.3 |  |  |
| Self-employed | 45 | 45.5 |  |  | 36 | 36.4 |  |  | 21 | 21.0 |  |  |
| Economically inactive | 106 | 38.4 |  |  | 70 | 25.4 |  |  | 59 | 21.3 |  |  |
| Not working | 44 | 55.7 |  |  | 35 | 44.3 |  |  | 24 | 30.4 |  |  |
| Disability / long-term illness | 57 | 46.0 |  |  | 35 | 28.2 |  |  | 38 | 30.6 |  |  |
| **Education** |  |  |  |  |  |  |  |  |  |  |  |  |
| GCSE / O-Level | 62 | 45.3 | **0.024** | A-level v No qualifications | 29 | 21.3 | **0.005** | Vocational v No qualifications;  Bachelor v No qualifications | 33 | 24.1 | 0.06 |  |
| Vocational | 30 | 47.6 |  |  | 25 | 39.7 |  |  | 25 | 39.7 |  |  |
| A level or equivalent | 117 | 50.9 |  |  | 77 | 33.5 |  |  | 56 | 24.3 |  |  |
| Bachelor’s degree or equivalent | 118 | 46.5 |  |  | 85 | 33.5 |  |  | 71 | 28.0 |  |  |
| Postgraduate degree or equivalent | 40 | 37.4 |  |  | 34 | 31.5 |  |  | 25 | 23.1 |  |  |
| Other | 31 | 45.6 |  |  | 20 | 29.4 |  |  | 12 | 17.6 |  |  |
| No formal qualifications | 40 | 32.0 |  |  | 23 | 18.4 |  |  | 25 | 20.0 |  |  |

^2^ Full-time – employed 30+ hours; Part-time – employed 29 hours or less; Economically inactive – full time students, still at school, retired or not in paid work due to long-term illness or disability; Not working – Unemployed and seeking work, Not in paid work for other reasons, or not working – housewife.

## Table S11c. Variations in responses desired (disciplinary action, investigation, financial compensation) by the NHS following harm by sociodemographic factors and harm through access or lack of access to treatment or care (* post hoc analysis reported for significant results (p<0.05))

|  | **Disciplinary action** | | | | **An investigation** | | | | **Financial compensation** | | | |
| --- | --- | --- | --- | --- | --- | --- | --- | --- | --- | --- | --- | --- |
|  | **Yes** | | | | **Yes** | | | | **Yes** | | | |
|  | **n** | **%** | **p** | **Post-hoc*** | **n** | **%** | **p** | **Post-hoc*** | **n** | **%** | **p** | **Post-hoc*** |
| **Sex** |  |  |  |  |  |  |  |  |  |  |  |  |
| Male | 28 | 7.9 | 0.07 |  | 68 | 19.1 | 0.24 |  | 30 | 8.5 | 0.79 |  |
| Female | 71 | 11.6 |  |  | 137 | 22.3 |  |  | 55 | 9.0 |  |  |
| **Age** |  |  |  |  |  |  |  |  |  |  |  |  |
| 18-24 | 9 | 7.2 | **0.006** | 35-44 v 65+ | 29 | 23.2 | 0.42 |  | 5 | 4.0 | 0.07 |  |
| 25-34 | 21 | 11.7 |  |  | 41 | 23.0 |  |  | 18 | 10.1 |  |  |
| 35-44 | 28 | 16.0 |  |  | 41 | 23.4 |  |  | 21 | 12.0 |  |  |
| 45-54 | 18 | 10.1 |  |  | 39 | 21.9 |  |  | 18 | 10.1 |  |  |
| 55-64 | 16 | 10.6 |  |  | 27 | 17.9 |  |  | 15 | 9.9 |  |  |
| 65+ | 7 | 3.9 |  |  | 29 | 16.1 |  |  | 9 | 5.0 |  |  |
| **Social grade ^1^** |  |  |  |  |  |  |  |  |  |  |  |  |
| A | 0 | 0 | Not run |  | 3 | 10.7 | **0.010** | C1 v D  D v E | 2 | 7.1 | Not run |  |
| B | 15 | 8.8 |  |  | 31 | 18.0 |  |  | 12 | 7.0 |  |  |
| C1 | 22 | 9.2 |  |  | 58 | 24.5 |  |  | 16 | 6.8 |  |  |
| C2 | 21 | 11.0 |  |  | 45 | 23.6 |  |  | 13 | 6.8 |  |  |
| D | 13 | 9.1 |  |  | 16 | 11.1 |  |  | 13 | 9.1 |  |  |
| E | 19 | 12.3 |  |  | 38 | 24.7 |  |  | 20 | 13.0 |  |  |
| **Access / lack of access** |  |  |  |  |  |  |  |  |  |  |  |  |
| Harmed through the lack of access | 16 | 4.4 | **<0.001** |  | 55 | 15.2 | **<0.001** |  | 16 | 4.4 | **<0.001** |  |
| Harmed by treatment or care | 83 | 13.5 |  |  | 152 | 24.8 |  |  | 69 | 11.2 |  |  |

^1^ Social grade: National Readership Survey Classification A (highest grade) to E (lowest grade) <https://nrs.co.uk/nrs-print/lifestyle-and-classification-data/social-grade/>

## Table S11c (continued). Variations in responses desired (disciplinary action, investigation, financial compensation) by the NHS following harm by sociodemographic factors and harm through access or lack of access to treatment or care (* post hoc analysis reported for significant results (p<0.05))

|  | **Disciplinary action** | | | | **An investigation** | | | | **Financial compensation** | | | |
| --- | --- | --- | --- | --- | --- | --- | --- | --- | --- | --- | --- | --- |
|  | **Yes** | | | | **Yes** | | | | **Yes** | | | |
|  | **n** | **%** | **p** | **Post-hoc*** | **n** | **%** | **p** | **Post-hoc*** | **n** | **%** | **p** | **Post-hoc*** |
| **Employment ^2^** |  |  |  |  |  |  |  |  |  |  |  |  |
| Employed full-time | 25 | 8.9 | **<0.001** | Employed v disability  PT v econ inactive  PT v disability  Econ inactive v not working  Econ inactive v disability | 58 | 20.6 | **<0.001** | PT v disability  Econ inactive v not working  Econ inactive v disability | 19 | 6.8 | **<0.001** | *FT v disability*  *Econ inactive v not working*  *Econ inactive v disability* |
| Employed part-time | 17 | 13.8 |  |  | 18 | 14.6 |  |  | 11 | 8.9 |  |  |
| Self-employed | 9 | 9.1 |  |  | 20 | 20.2 |  |  | 9 | 9.1 |  |  |
| Economically inactive | 10 | 3.6 |  |  | 44 | 15.9 |  |  | 11 | 4.0 |  |  |
| Not working | 12 | 15.2 |  |  | 25 | 31.6 |  |  | 13 | 16.3 |  |  |
| Disability / long-term illness | 26 | 21.0 |  |  | 40 | 32.3 |  |  | 22 | 17.7 |  |  |
| **Education** |  |  |  |  |  |  |  |  |  |  |  |  |
| GCSE / O-Level | 9 | 6.6 | 0.08 |  | 25 | 18.4 | 0.61 |  | 10 | 7.4 | 0.33 |  |
| Vocational | 9 | 14.3 |  |  | 18 | 28.6 |  |  | 5 | 7.9 |  |  |
| A level or equivalent | 31 | 13.5 |  |  | 45 | 19.6 |  |  | 23 | 10.0 |  |  |
| Bachelor’s degree or equivalent | 21 | 8.3 |  |  | 60 | 23.6 |  |  | 19 | 7.5 |  |  |
| Postgraduate degree or equivalent | 8 | 7.5 |  |  | 22 | 20.6 |  |  | 9 | 8.4 |  |  |
| Other | 3 | 4.4 |  |  | 13 | 18.8 |  |  | 2 | 2.9 |  |  |
| No formal qualifications | 16 | 12.9 |  |  | 24 | 19.2 |  |  | 16 | 12.8 |  |  |

^2^ Full-time – employed 30+ hours; Part-time – employed 29 hours or less; Economically inactive – full time students, still at school, retired or not in paid work due to long-term illness or disability; Not working – Unemployed and seeking work, Not in paid work for other reasons, or not working – housewife.
