# Supplementary material for: APPROACH: Analysis of Proton versus Photon Radiotherapy in Oligodendroglioma and Assessment of Cognitive Health – study protocol paper for a phase III multicentre, open-label randomised controlled trial
Source: BMJ Open. 2025 Feb 26;15(2):e097810. doi: 10.1136/bmjopen-2024-097810 (PMC11865786; doi:10.1136/bmjopen-2024-097810)
Supplement: Supplementary file 2 [file bmjopen-2024-097810.reviewer_comments.pdf]

PEER REVIEW HISTORY

BMJ Open publishes all reviews undertaken for accepted manuscripts. Reviewers are asked to complete a checklist review form and are provided with free text boxes to elaborate on their assessment. These free text comments are reproduced below.

ARTICLE DETAILS

Title (Provisional)

APPROACH: Analysis of Proton versus Photon Radiotherapy in Oligodendroglioma and Assessment of Cognitive Health: Study protocol paper for a phase III multicentre, open-label randomised controlled trial

Authors

Slevin, Finbar; Hudson, Eleanor Mae; Boele, Florien W.; Powell, James R.; Noutch, Samantha; Borland, Myfanwy; Brown, Sarah; Bruce, Anna; Bulbeck, Helen; Burnet, Neil G.; Chang, Yen Ching; Colaco, Rovel; Currie, Stuart; Egleston, Daniel; Fersht, Naomi; Klein, Martin; Lilley, John; Lowe, Matthew; Miles, Elizabeth; Murray, Robert D.; O’Hara, Daniel J.; Norris, Matthew; Parbutt, Catherine; Smith, Alexandra; Smith, Charlotte; Whitfield, Gillian A.; Short, Susan; Murray, Louise

---

This article was not externally reviewed at BMJ Open. Protocols that have been independently assessed prior to submission to BMJ Open are usually fast-tracked to publication on the grounds that further substantial changes will not be possible. This independent assessment will usually be external, independent review for both a substantial grant award from a non-commercial or government funder and ethics approval.
